# Interleukin‐33 increases type 2 innate lymphoid cell count and their activation in eosinophilic asthma

**DOI:** 10.1002/clt2.12265

**Published:** 2023-06-01

**Authors:** Fengfei Sun, Wei Zou, Honglei Shi, Zehu Chen, Donghai Ma, Minmin Lin, Kongqiu Wang, Yiying Huang, Xiaobin Zheng, Cuiyan Tan, Meizhu Chen, Changli Tu, Zhenguo Wang, Jian Wu, Weiming Wu, Jing Liu

**Affiliations:** ^1^ Department of Pulmonary and Critical Care Medicine the Fifth Affiliated Hospital of Sun Yat‐sen University Zhuhai China; ^2^ Guangdong Provincial Key Laboratory of Biomedical Imaging and Guangdong Provincial Engineering Research Center of Molecular Imaging the Fifth Affiliated Hospital of Sun Yat‐sen University Zhuhai China; ^3^ Department of Allergy the Fifth Affiliated Hospital of Sun Yat‐sen University Zhuhai China

**Keywords:** bronchial asthma, eosinophils, interleukin‐33, type 2 innate lymphoid cell

## Abstract

**Background:**

Interleukin‐33 (IL‐33) exacerbates asthma probably through type 2 innate lymphoid cells (ILC2s). Nevertheless, the association between eosinophilic asthma (EA) and ILC2s remains obscure, and the mechanisms by which IL‐33 affects ILC2s are yet to be clarified.

**Methods:**

ILC2s were evaluated in peripheral blood mononuclear cells, induced sputum, and bronchoalveolar lavage fluid obtained from patients with EA. Confocal microscopy was performed to locate ILC2s in lung tissue and the mRNA expression of ILC2‐related genes was also evaluated in the EA model. The proliferation of ILC2s isolated from humans and mice was assessed following IL‐33 or anti‐IL‐33 stimulation.

**Results:**

The counts, activation, and mRNA expression of relevant genes in ILC2s were higher in PBMCs and airways of patients with EA. In addition, ILC2 cell counts correlated with Asthma control test, blood eosinophil count, Fractional exhaled nitric oxide level, and predicted eosinophilic airway inflammation. IL‐33 induced stronger proliferation of ILC2s and increased their density around blood vessels in the lungs of mice with EA. Moreover, IL‐33 treatment increased the counts and activation of ILC2s and lung inflammatory scores, whereas anti‐IL‐33 antibody significantly reversed these effects in EA mice. Finally, IL‐33 enhanced PI3K and AKT protein expression in ILC2s, whereas inhibition of the PI3K/AKT pathway decreased IL‐5 and IL‐13 production by ILC2s in EA.

**Conclusions:**

ILC2s, especially activated ILC2s, might be critical markers of EA. IL‐33 can induce and activate ILC2s in the lungs via the PI3K/AKT pathway in EA. Thus, using anti‐IL‐33 antibody could be a part of an effective treatment strategy for EA.

## 1 INTRODUCTION

Asthma, a prevalent chronic airway illness, is classified into the eosinophilic asthma (EA), neutrophilic asthma, mixed asthma, and paucigranulocytic asthma based on the counts of eosinophils and neutrophils in the sputum. Patients with EA exhibit worse symptoms than those with non‐EA (non‐eosinophilic asthma (NEA)), regardless of asthma severity.^1^ Recent studies have confirmed the crucial involvement of innate lymphoid cells (ILC2s), a rare group of lymphocytes, in innate immune responses to allergic diseases.^2^

Innate lymphoid cells lack antigen recognition receptors and produce IL‐13 and IL‐5, both of which are involved in EA pathogenesis.^3^ Interleukin‐33, which is produced by bronchial epithelial cells, is crucial for the activation of resident lung Th2 cells as well as ILC2s to produce IL‐5, resulting in the development of chitin‐induced airway eosinophilia.^4^ In addition, IL‐33 and IL1RL1 (ST2) variants are reported to be significantly associated with asthma.^5^, ^6^, ^7^ Studies have established the correlation of a higher number of ILC2s in the blood, sputum, or bronchoalveolar lavage fluid (BALF) in allergic asthma, and the increased number of ILC2s has been related to asthma exacerbation.^8^, ^9^, ^10^ In the lungs, ILC2s, along with dendritic cells and regulatory T cells, localize to the lung bronchi and larger vessels.^11^ However, the localization of ILC2s in the lungs has been mostly studied in transgenic mice, which limits the interpretation of the results and their application to humans. Moreover, only a limited number of studies have documented the association between EA and ILC2s.

In patients with allergic asthma, increased levels of ILC2s are linked to increased eosinophils.^12^ IL‐33 is crucial for the activation of ILC2s.^13^ Notably, the p38 mitogen‐activated protein kinase (MAPK), NF‐κB, PI3K/Akt, and Wnt/β‐catenin pathways regulate the transcription of IL‐33 in murine and human endothelial cells^14^ and activation of the PI3K/AKT signaling pathway induces biological processes in eosinophils.^15^ Therefore, additional studies are required to address the mechanisms of IL‐33 in ILC2s in EA. This study aimed to assess the level and function of ILC2s in patients with EA as well as their correlation with IL‐33.

## 2 METHODS

### 2.1 Study design and subjects

This study was approved by the Ethics Committee of the Fifth Affiliated Hospital of Sun Yat‐sen University (approval no. K35‐1). Informed consent was obtained from 255 patients with asthma and 54 healthy controls (HCs). After applying inclusion and exclusion criteria, 203 patients with asthma and 40 healthy individuals were enrolled in the study. Supplementary Figure S1 shows a detailed description of the research design.

### 2.2 Murine model of eosinophilic asthma

We randomly divided C57BL/6J mice of 6–8‐week‐old, in Guangdong Medical Laboratory Animal Center (Zhuhai, Guangzhou, China) into four groups (*n* = 6): negative control (NC) (Equivalent volume of phosphate buffer saline), ovalbumin (OVA) (20 μg OVA+100 μL alum on day 0 and 14 i.p, ultrasonic nebulization with 1% OVA on days 24, 25, and 26), OVA+IL33 (rIL33 0.5 μg intranasally once every other day, three times in total), and OVA + anti‐IL33 (anti‐IL33 antibody (50 mg i.p for six consecutive days). A detailed description is presented in the supplementary material.

### 2.3 Induced sputum and classification of inflammatory cells

Sputum specimens were obtained as previously reported.^16^ Individuals with ≥3% eosinophils in sputum were considered to have EA. A detailed description is provided in the supplementary material.

### 2.4 Evaluation of ILC2s using flow cytometry

Briefly, 1 × 10^6^ PBMCs, sputum cells, or BALF cells were incubated with BV‐421‐CD45, FITC‐lineage cocktail, APC‐CD127, or phycoerythrin (PE)‐CD294 antibodies (above all of antibodies from BioLegend, San Diego, CA, USA) for the detection of ILC2s. To assay the intracellular levels of IL‐5 and IL‐13, cells were cultured for 4 h in RPMI‐640 medium (Invitrogen, California, USA) containing 10% fetal bovine serum (Invitrogen, California, USA), 50 ng/mL phorbol‐12‐myristate‐13‐acetate (Sigma‐Aldrich, Shanghai, China), 1 μg/mL ionomycin (Sigma‐Aldrich, Shanghai, China), and 1 μg/mL brefeldin A (BioLegend, San Diego, CA, USA). Subsequently, PE/Cyaine7‐IL‐13 and Briliant Violet 650‐IL‐5 antibodies (BioLegend, San Diego, CA, USA) were used to detect IL‐5+ILC2s and IL‐13+ILC2s, respectively. Samples were subjected to flow cytometry (BD laser II) and analyzed using the Kaluza software (Beckman, BD, USA).

### 2.5 Pathological staining and inflammatory cell count

Mice lung tissue embedded in paraffin were sliced, dewaxed, dehydrated in a graded series of ethanol, and stained with hematoxylin‐eosin staining. Bronchus‐bronchial lesions were assessed and scored under a light microscope according to a previous method.^17^ A detailed description is provided in the supplementary material.

### 2.6 Enzyme‐linked immunosorbent assay

IL‐5, IL‐33 and IgE in the plasma of the subject and IL‐5, IL‐13 and OVA‐IgE in the culture supernatant of ILC2s isolated from lung tissue in mice were determined using ELISA kits (MEIMIAN, Wuhan, China) according to the manufacturer's protocol.

### 2.7 Location of ILC2s in lung tissue using confocal microscopy

Briefly, 4‐μm‐thick murine lung tissue slices were dewaxed and rehydrated in a graded series of ethanol. Next, their endogenous peroxidase activity was blocked and the sections were subjected to epitope retrieval, following which, they were incubated with primary antibodies against Anti‐mouse CD3e‐biotin (dilution 1:100 eBio500A2, eBioscience,Cal, USA), Anti‐rat GATA3 (dilution 1:50, ab110093, Abcam, Mass, USA), and Anti‐rabbit inducible T‐cell costimulatory (ICOS) (dilution 1:200, ab175401, Abcam, Mass, USA). Bound biotinylated antibodies were detected using streptavidin‐AF555 conjugated secondary antibodies (S32355, Invitrogen, Carlsbad, Cal, USA), then anti‐GATA3 antibodies were detected using rabbit anti‐rat IgG‐FITC‐AF488 (A‐11006, Invitrogen, Carlsbad, Cal, USA). Finally, ICOS antibodies were detected using goat anti‐rabbit IgG‐647 secondary antibodies (A‐27040, Invitrogen, Carlsbad, Cal, USA). Sections were counterstained with 4’,6‐Diamidino‐2’‐phenylindole (62248, Invitrogen, Carlsbad, Cal, USA). Images were acquired under an Laser Scanning Confocal Microscope (LSM) 510 meta confocal microscope (Zeiss, Oberkochen,UK) and the Zeiss LSM software was used for image analysis.

### 2.8 Evaluation of ILC2‐related gene expression using RT‐qPCR

Total RNA was extracted from human peripheral blood or mouse lung tissue using TRIzol LS (Thermo Fisher Scientific, Waltham, MA, USA). A detailed description and primer sequences are provided in the supplementary material.

### 2.9 Sorting of ILC2s from human PBMCs and murine lung tissues and evaluation of proliferation of ILC2 in vitro

PBMCs were isolated from individuals in the HC and EA groups. In addition, lungs were resected from mice in the NC and EA groups. Innate lymphoid cells were sorted using magnetic beads (STEMCELL, BC, Canada); this process involved the isolation of CRTH2+ cells using column‐free immunomagnetic‐positive sorting. Finally, an ILC2 isolation cocktail was added, and the enriched cell suspension was transferred to a fresh tube using a magnet; non‐ILC2s were discarded. The process of ILC2 sorting in the lungs of mice is described in the supplementary material. Sorted ILC2s were cultured and relative cell viability was calculated as reported previously.^18^ A detailed description is provided in the supplementary material.

### 2.10 Western blot analysis

Innate lymphoid cells were sorted from the OVA+IL‐33 group and exposed to the AKT inhibitor AZD5363 (10 μmol/L; Selleck, Shanghai, China) for 24 h. A detailed description is provided in the supplementary material.

### 2.11 Statistical analyses

SPSS version 10.0(IBM, USA) was used for data analysis. Data are indicated as the mean ± SEM. In the subject experiment, the Kruskal‐Wallis test was used to compare multiple groups with uneven variance. In animal experiments, the one‐way analysis of variance (ANOVA) was used to compare multiple groups with equal variance. Count data were analyzed using X^2^ test. Spearman's rank correlation was used to analyze the correlations between ILC2s and other factors. Mann‐Whitney test was used to analyze the lung function index. Statistical significance was set at *p* < 0.05.

## 3 RESULTS

### 3.1 Number and associated gene expression of ILC2s increased in EA

**FIGURE 1:**
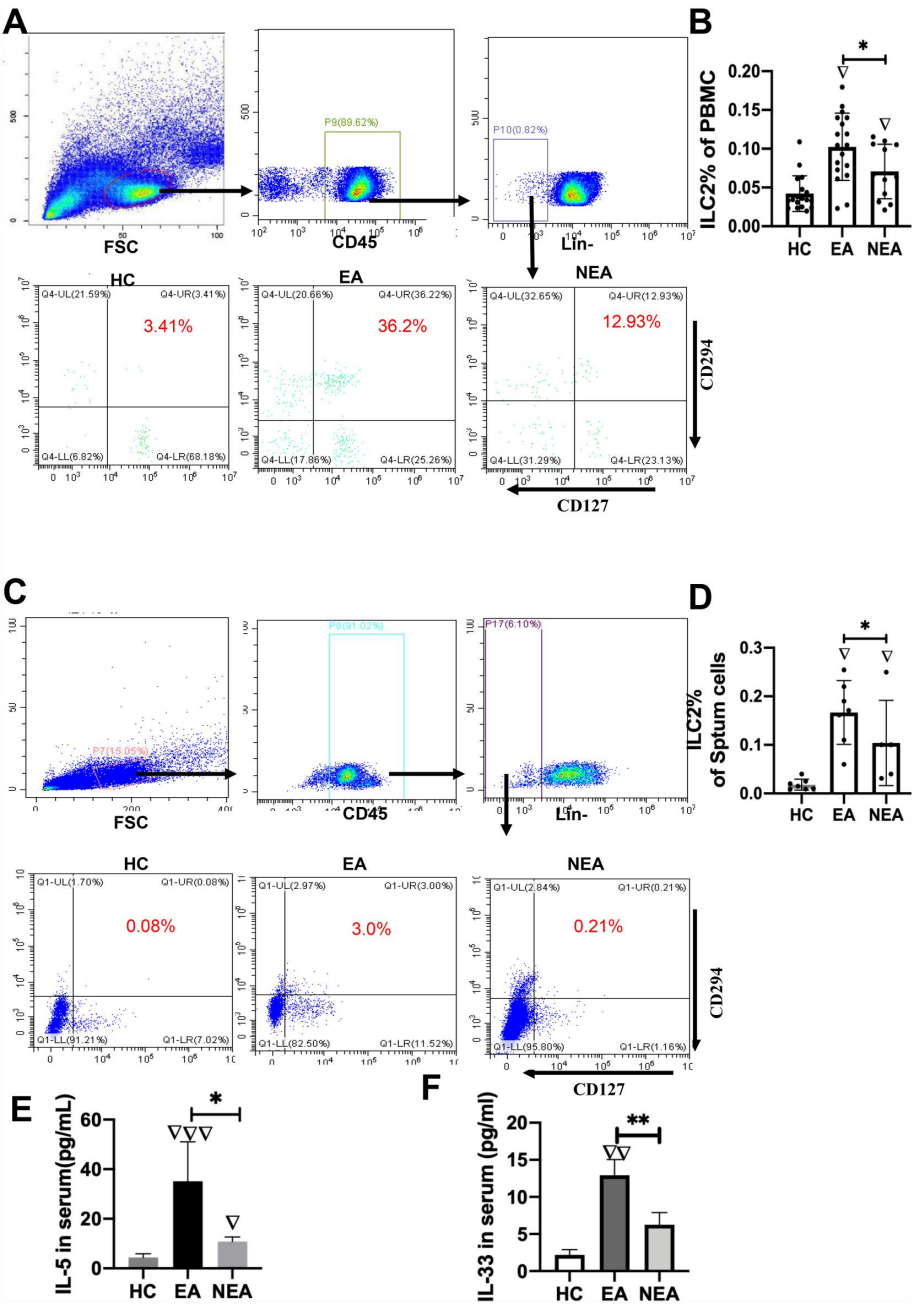
The innate lymphoid cell (ILC2) numbers were elevated in EA (A) ILC2s were gated as the CD45^+^Lin^−^CD127^+^CD294^+^ population in PBMCs obtained from HC and patients with asthma. (B) The numbers of ILC2s were significantly higher in PBMCs obtained from the EA group (*n* = 30) than those from the HC and NEA groups. (C) ILC2s were gated as the CD45^+^Lin^−^CD127^+^CD294^+^ population in induced sputum (IS) obtained from the HC group and patients with asthma. (D) The number of ILC2s was significantly higher in the IS of the EA group. (E–F) The levels of IL‐5 and IL‐33 were significantly higher in the serum of the EA group. HC, healthy control; EA, eosinophilic asthma; NEA, non‐eosinophilic asthma. ▽Comparison with HC group, ▽*p <* 0.05, ▽▽*p <* 0.01; ▽▽▽*p <* 0.001; *Comparison with EA group, *p < 0.05, **p < 0.01. The Kruskal‐Wallis test was used to analyze statistical significance between groups.

Innate lymphoid cells have been reported to express CRTH2 and CD127.^19^ In our study, ILC2s isolated from PBMCs and induced sputum (IS) of patients with asthma were sorted into the CD45^+^Lin^−^CD127^+^CRTH2^+^ population (Figure 1A–D). In particular, patients with asthma had higher numbers of ILC2s in PBMCs and sputum than the HC group (*p* < 0.05). Moreover, patients with EA had higher counts of ILC2s than those with NEA (*p* < 0.05, Figure 1B,D) and higher levels of IL‐5 and IL‐33 than those with NEA (*p* < 0.05, Fig. E and F). Furthermore, an increased proportion of IL‐5^+^ILC2s and IL‐13^+^ILC2s was observed in PBMCs of patients with asthma, particularly in the EA group (Figure S2). We also detected that the expression of *GATA3* and *RORa* was remarkably higher in the EA group compared with that in other groups (Figure S3A, B). However, *CD127* expression did not differ between the groups (Figure S3C). The *CRTH2* and *ICOS* mRNA levels were the highest in the EA group (PBMCs, Figure S3D, E), while *KLRG1* expression was increased in the EA group (Figure S3F). Finally, the *IL‐*33 mRNA level in the blood of EA patients and lung tissue of EA mice was remarkably higher than those in other groups (Figure S3G, Figure S7F).

### 3.2 Number of ILC2s correlated with ACT, blood eosinophil count, and FeNO levels, and predicted eosinophilic airway inflammation

**FIGURE 2:**
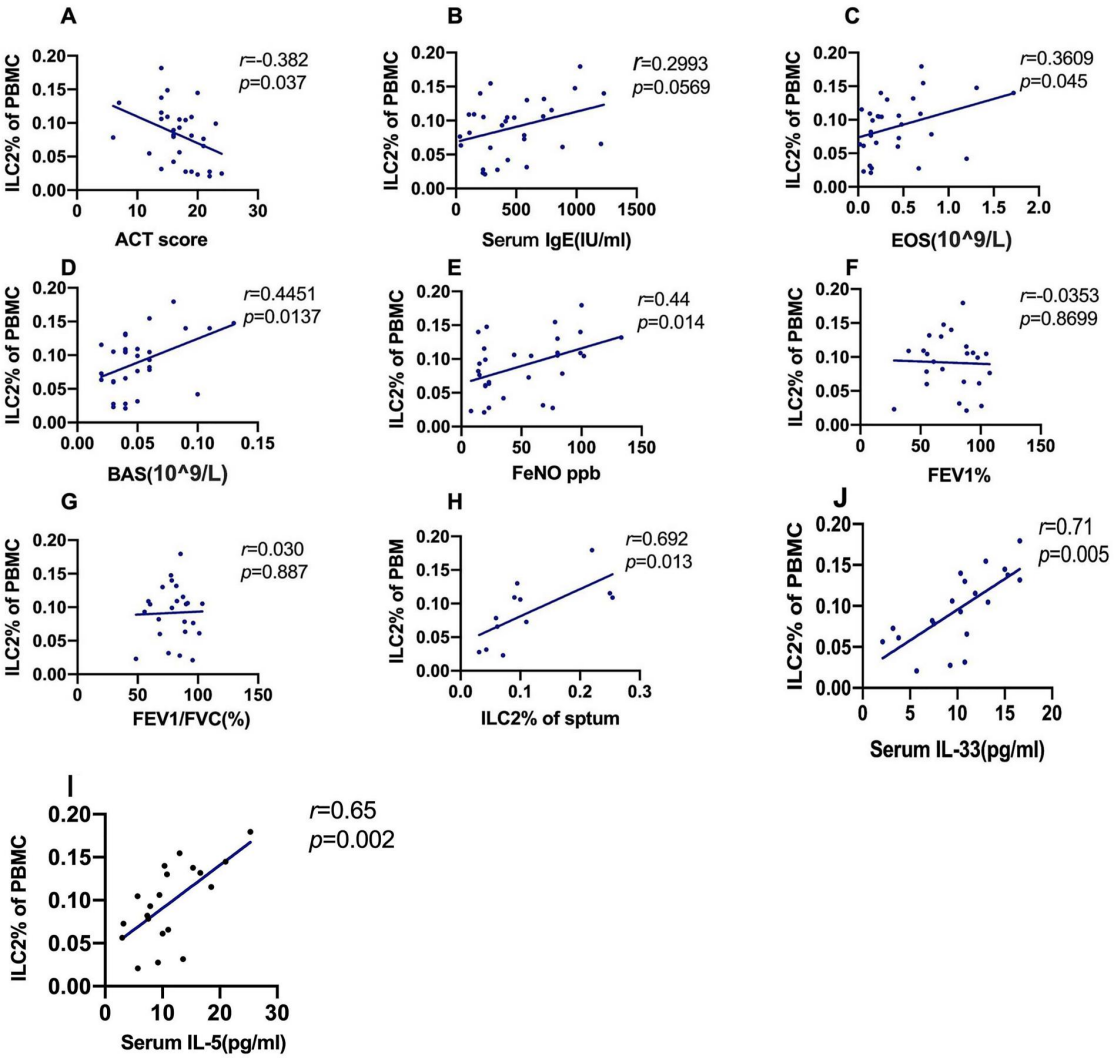
Correlation analysis between relevant factors and number of innate lymphoid cells (ILC2s) in patients with asthma. Correlation between the percentage of ILC2s in PBMCs and (A) Asthma control test (ACT) score, (B) serum IgE, (C) EOS in blood, (D) BAS in blood, (E) Fractional exhaled nitric oxide (FeNO), (F) FEV1% pred, (G) FEV1/FVC (%), (H) ILC2% of sputum, (J) serum IL‐33, and (I) serum IL‐5. EOS, eosinophils; BAS, basophils. Spearman's rank correlation was used to analyze the correlation between relevant factors and number of ILC2s. FVC, forced vital capacity.

Table 1 shows the baseline characteristics of the individuals enrolled in our study. The EA group had the highest counts of blood eosinophils and basophils (BASs). In addition, the EA group was characterized by a higher incidence of allergy history and omalizumab usage, increased Fractional exhaled nitric oxide (FeNO) level, and specific and total IgE, but lower Asthma control test (ACT) scores than those in other groups (Table 1). The EA group presented the lowest FEV1%pred and the highest Rc, Rp, Z5, R5, R20, and Fres among the four groups (Table S1 and Figure S4). Furthermore, the percentage of ILC2s in PBMCs positively correlated with the counts of blood eosinophils (*r*_*s*_ = 0.361, *p* = 0.045), blood BASs (*r*_*s*_ = 0.445, *p* = 0.0137), sputum ILC2s (*r*_*s*_ = 0.692, *p* = 0.013), levels of FeNO (*r*_*s*_ = 0.44, *p* = 0.014), serum IL‐5 (*r*_*s*_ = 0.65, *p* = 0.002), and serum IL‐33 (*r*_*s*_ = 0.71, *p* = 0.005), but negatively correlated with ACT (*r*_*s*_ = 0.382, *p* = 0.037). We did not detect any correlation between IgE, FEV1%, and FEV1/forced vital capacity (%) (*p* > 0.05) (Figure 2). Furthermore, using receiver operating characteristic analysis, we identified biomarkers of respiratory eosinophilic inflammation (sputum eosinophil count ≥3%). We found that ILC2s number was a sensitive EA diagnostic biomarker. Using a threshold value of ≥0.065% for ILC2s and ≥0.465 × 10^9^ for eosinophils, we could distinguish between eosinophilic and non‐eosinophilic inflammation with high sensitivity and specificity (89% and 50%, respectively, Table S2). Moreover, the area under the curve of blood eosinophils and FeNO for differentiating between eosinophilic and non‐eosinophilic inflammation was 0.708 and 0.741, respectively (*p* = 0.05, *p* = 0.028; Figure S5) However, IgE level and ACT score could not distinguish between eosinophilic and non‐eosinophilic inflammation (Table S2). These factors were selected based on the Spearman rank test.

**TABLE 1:** Characteristics of patients with asthma with different inflammatory phenotypes.

	Control	Eosinophilic phenotype	Neutrophilic phenotype	Mixed granulocytic phenotype	Paucigranulocytic phenotype	*p* value
*n*	40	98 (46.4%)	25 (17.14%)	23 (13.57%)	57 (22.86%)	0.055
Sex (male) *n* (%)	21 (52.5)	31 (48.44)	13 (46.43)	4 (50)	26 (52)	0.26
Age	40.23 ± 1.64	49.59 ± 1.93*	53.85 ± 3.16*	58.12 ± 2.87*^#^	46.12 ± 2.99	0.034
BMI	23.45 ± 0.23	23.65 ± 0.46	23.30 ± 0.71	23.30 ± 0.94	24.29 ± 0.891	0.172
ACT scores	25 (25/25)	15.69 ± 0.22*	18.08 ± 0.68*	18.60 ± 0.81*^#^	19.99 ± 0.23^#^	0.000
SIgE(+) *n* (%)	0	41 (42)*	5 (20)*^#^	6 (26)*^#^	14 (24.6)*^#^	0.000
Total IgE (IU/mL)	26.78 (12.73/54.89)	483.2 (242.8/950.6)*	94.46 (29.43/227.3)*^#^	375 (105.5/680.8)*	65.12 (32.32/262.3)*^#^	0.000
Smoking *n* (%)	10 (14)	16 (20)	7 (24.1)	6 (26)	11 (19.3)	0.542
Allergic rhinitis *n* (%)	0	30 (34.1)*	3 (12)*^#^	9 (39.1)*	20 (35.1)*	0.038
Allergic history *n* (%)	0	50 (51)*	6 (24)*^#^	12 (52.2)*^&^	15 (26.3)*^#^	0.05
Diseases history (years)	0	7.54 ± 1.09	7.91 ± 2.46	7.83 ± 2.29	7.24 ± 1.25	0.001
Chronic disease *n* (%)	2 (5)	16 (16.3)*	7 (28)*	4 (17.4)*	11 (19.3)*	0.03
Omalizumab *n* (%)	0	29 (29.6)*^#^	2 (0.09)*^#^	2 (0.08)*^#^	0*^#^	0.000
Antibiotics *n* (%)	0	31 (31.6)*	13 (52)*^#^	9 (39.1)*	16 (28.1)*^&^	0.038
Inhaled remedies *n* (%)	0	72 (71.4)*	10 (40)*^#^	12 (52.2)*	15 (26.3)*^#^	0.000
Hospital day	0	5 (3/8)*	4.5 (3/7)*	4 (2/7)*	5 (3/8)*	0.000
BAS (×10^9^)	0.03 (0.01/0.04)	0.05 (0.03/0.07)*	0.03 (0.03/0.05)^#^	0.04 (0.02/0.07)*^#^	0.04 (0.02/0.06)*^#^	0.004
Lym (×10^9^)	2.04 (1.3/2.75)	1.94 (1.59/2.44)	1.98 (1.53/2.53)	1.71 (1.34/1.9)*^#&^	1.81 (1.4/2.08)*	0.02
NLR	2.1 (1.15/2.23)	2.09 (1.08/2.43)	2.22 (1.3/2.21)	3 (1.23/3.4)*^#&^	2.5 (1.34/2.7)*	0.047
EOS (×10^9^)	0.18 (0.09/0.22)	0.45 (0.18/0.73)*	0.15 (0.07/0.21)^#^	0.3 (0.16/0.45)*^#^	0.14 (0.06/0.24)^#^	0.000
EOS%	3.77 (1.82/5.81)	6.3 (2.85/10.43)*	2.15 (1.13/3.78)^#^	3.6 (2.8/6.8)*^#^	2.2 (0.95/3.3)^#^	0.000
FeNO (ppb)		51 (21/90)	25 (17/29)	33 (22.25/92.6)	19 (14/26.75)^#^	0.001

*Note*: Data are presented as either the mean ± SEM or median (25%–75%) IQR. *p < 0.05, compared with the control group; ^#^ *p <* 0.05, compared with eosinophilic asthma; and ^&^ *p <* 0.05, compared with the neutrophilic group. The Kruskal‐Wallis test was used to analyze statistical significance between groups. Count data were analyzed using X^2^ test.

Abbreviation: BAS, basophil; BMI, Body Mass Index; EOS, Eosinophil; IQR, interquartile range; NLR, Neutrophil to Lymphocyte ration.

### 3.3 IL‐33 induced proliferation and pulmonary accumulation of ILC2s in EA

**FIGURE 3:**
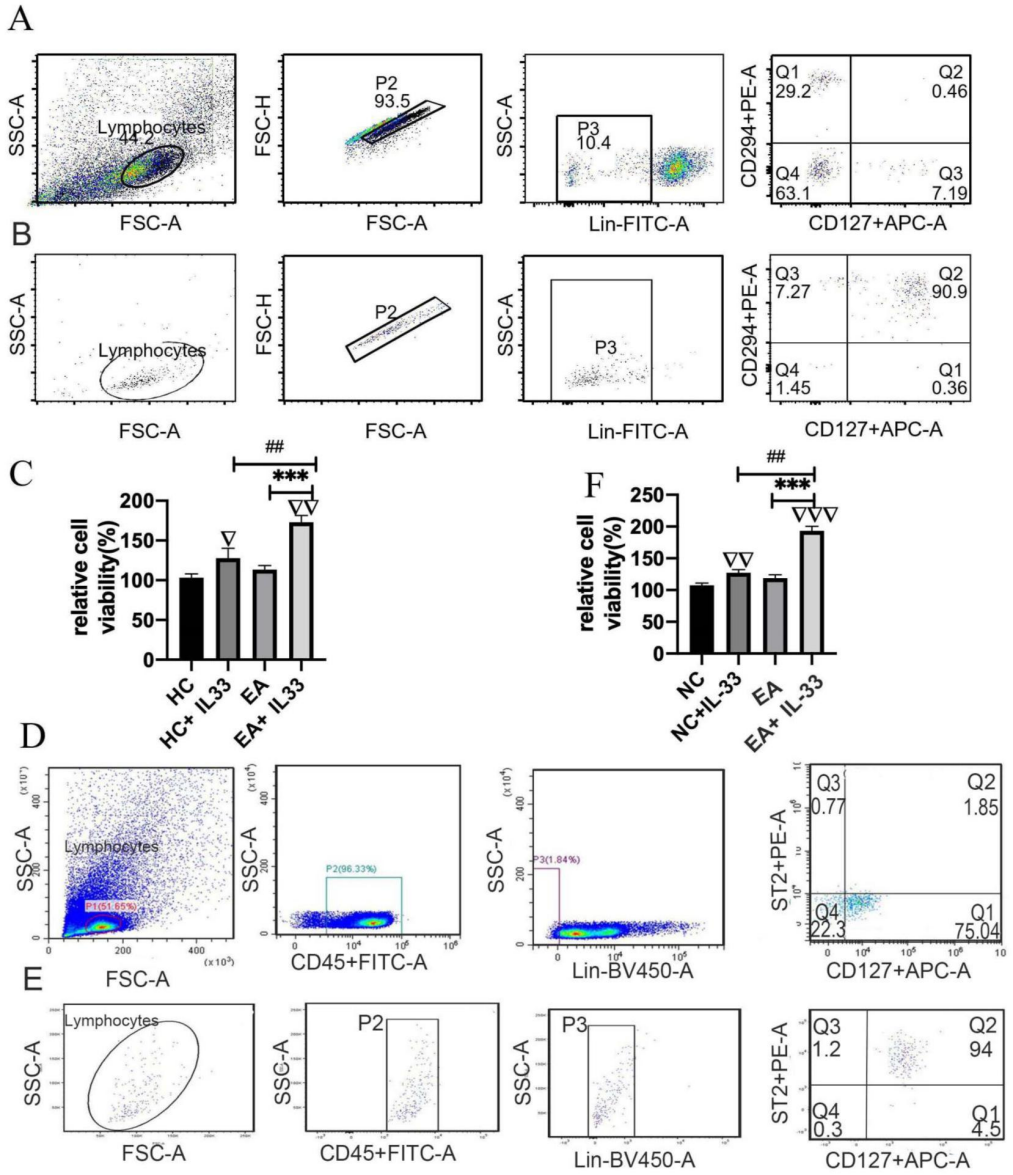
Innate lymphoid cells (ILC2s) in the EA group showed the strongest proliferation after IL‐33 stimulation in vitro. (A) ILC2s accounted for 0.46% of PBMCs before isolation from the peripheral blood of a patient with eosinophilic asthma. (B) The percentage of ILC2s reached up to 91% after isolation from a patient with eosinophilic asthma as determined by flow cytometry. (C) In vitro relative cell viability of ILC2s obtained from HC and patients with EA, with or without IL‐33 stimulation. (D) ILC2s accounted for 1.49% of cells present in lung homogenate before isolation in mice with eosinophilic asthma. (E) The percentage of ILC2s reached up to 94% after isolation from mice with eosinophilic asthma, as determined by performing flow cytometry. (F) Relative cell viability of ILC2s in vitro with or without IL‐33 stimulation in NC and EA mice. HC, healthy control; EA, eosinophilic asthma; NC, negative control. ▽Comparison with HC or NC group; ▽*p <* 0.05; ▽▽*p <* 0.01; ▽▽▽*p <* 0.001; *Comparison with EA group; ***p < 0.001; #Comparison with HC+IL‐33 or NC+IL‐33 group; *##P＜* 0.01. ANOVA test was used to analyze statistical significance between groups. ANOVA, analysis of variance.

**FIGURE 4:**
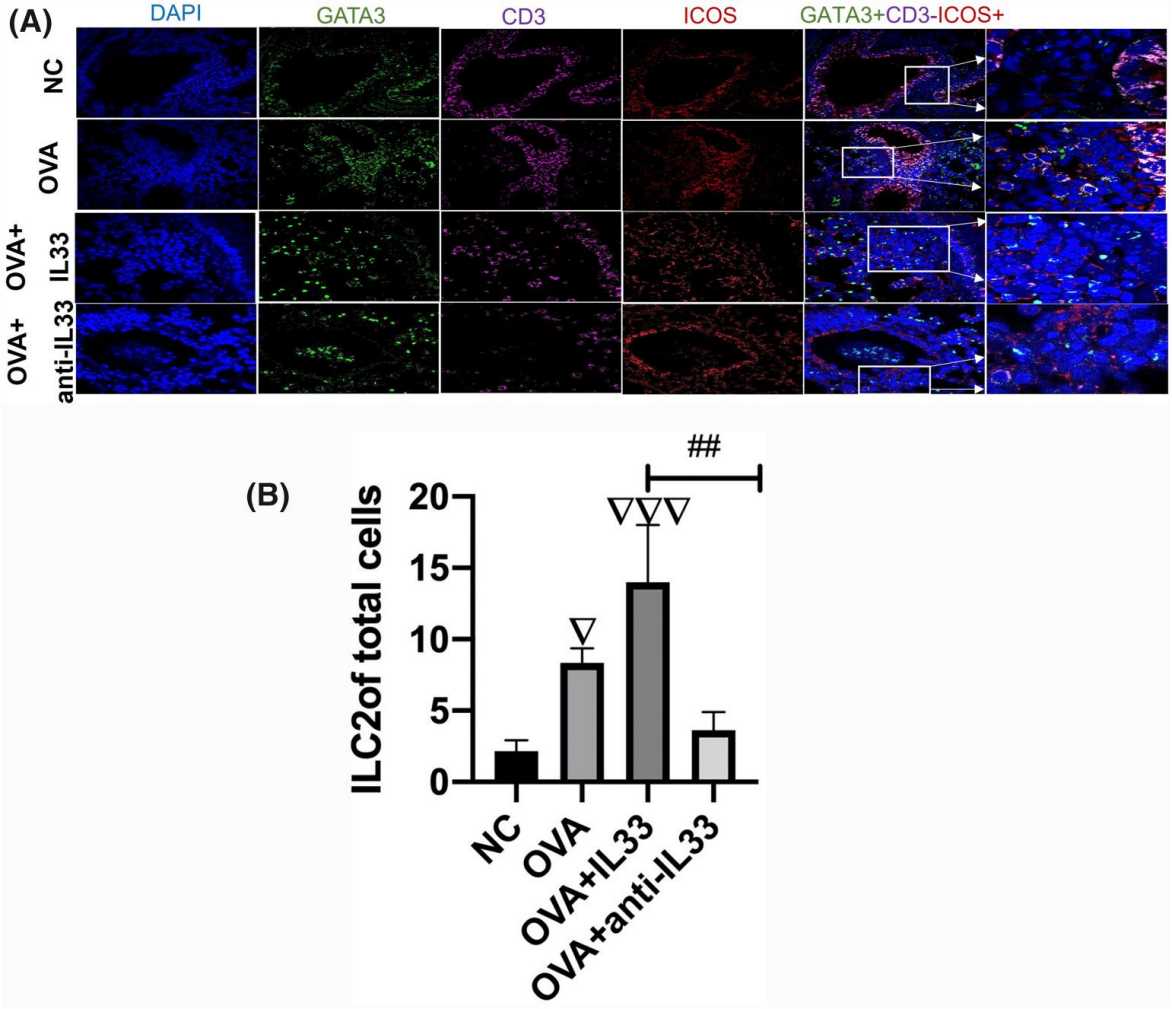
IL‐33 increased innate lymphoid cell (ILC2) cell accumulation around blood vessels in the lungs. (A) Localization of GATA3^+^CD3^−^ICOS^+^ ILC2s in the lungs of mice with asthma as determined by confocal microscopy. (B) The number of ILC2s in the lungs was highest in the OVA + IL‐33 group. NC, negative control. ▽Comparison with NC group; ▽*p <* 0.05; ▽▽▽*p <* 0.001; #Comparison with OVA + IL‐33 group*; ##p <* 0.01; *###p <* 0.001. ANOVA test was used to analyze statistical significance between groups. ANOVA, analysis of variance; OVA, ovalbumin; ICOS, inducible T‐cell costimulatory.

We isolated ILC2s from the peripheral blood of patients in the EA and HC groups at 91% purity (Figure 3A,B). In addition, we obtained ILC2s from the lung tissue of EA and NC mice with a purity of up to 94% (Figure 3D,E). We found that ILC2s from the control and EA groups proliferated rapidly when cocultured with rIL‐33 in vitro. However, we did not detect any difference in the in vitro proliferation of ILC2s obtained from the control and EA groups. Interestingly, ILC2s obtained from the EA group showed stronger in vitro proliferation after treatment with rIL‐33 compared with those from other groups (Figure 3C,D). We also examined the distribution and proliferation of ILC2s in vivo. We labeled and observed ILC2s (CD3‐GATA3+ICOS+) gathered around lung blood vessels using confocal microscopy and found an increased accumulation of ILC2s around the blood vessels of murine lungs in the EA group following rIL‐33 stimulation (Figure 4A,B).

### 3.4 IL‐33 enhanced the activation of IL‐5+ILC2s and IL‐13+ILC2s, the associated gene expression of ILC2s, and aggravated lung allergic inflammation in mice with EA

**FIGURE 5:**
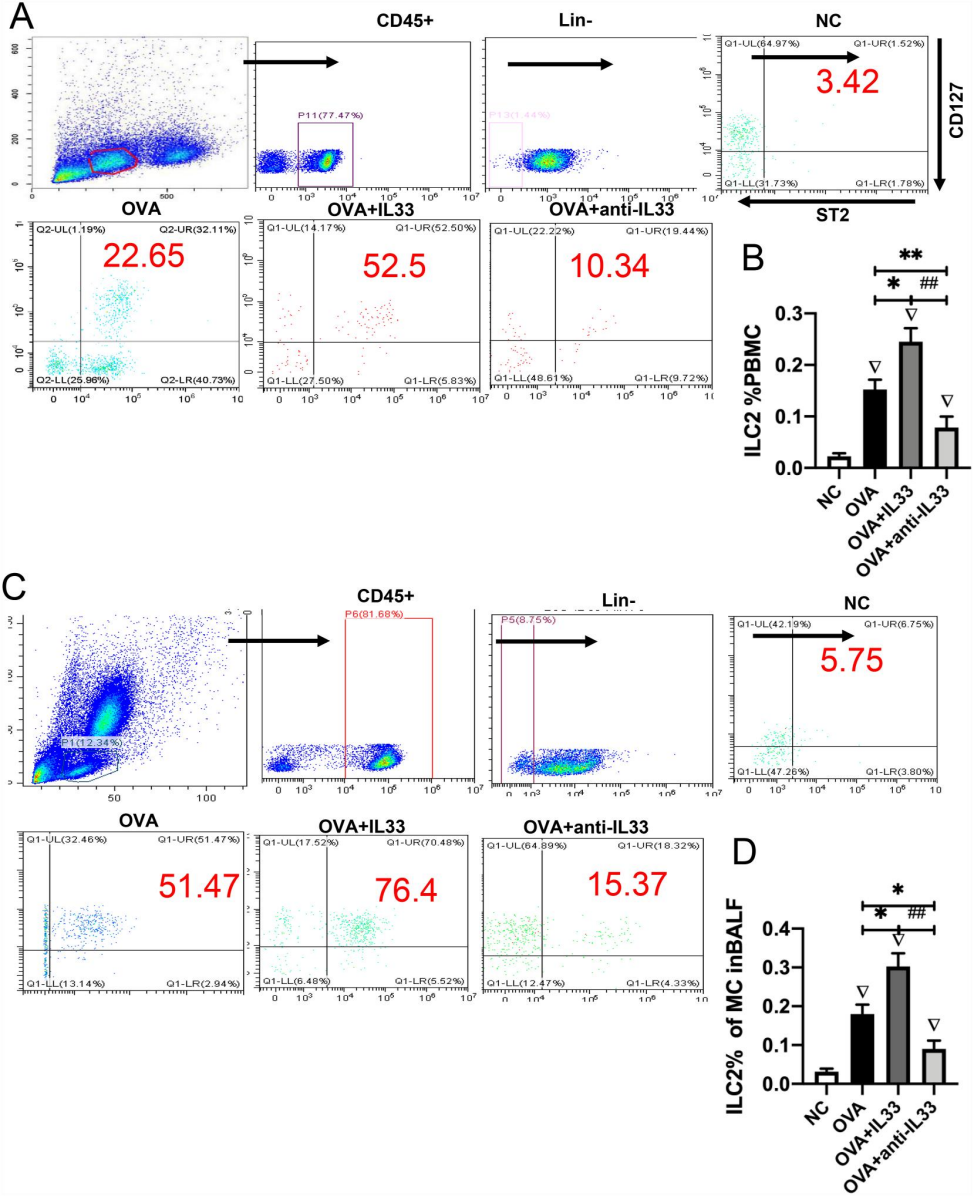
IL‐33 enhanced the number of innate lymphoid cells (ILC2s) in PBMCs and BALF of EA mice. (A) ILC2s were gated as the CD45^+^Lin^−^CD127^+^ST2^+^ population in PBMCs obtained from mice. (B) The number of ILC2s in PBMCs was highest in the OVA + IL‐33 group. (C) ILC2s were gated as the CD45^+^Lin^−^CD127^+^ST2^+^ population in BALF of mice. (D) The number of ILC2s in BALF was highest in the OVA + IL‐33 group. NC, negative control; EA, eosinophilic asthma; MC, mononuclear cell; BALF, bronchoalveolar lavage fluid. ▽Comparison with NC group, ▽*p <* 0.05; *Comparison with OVA group, *p < 0.05; **p < 0.01; *#*Comparison with OVA + IL‐33 group*; ##p <* 0.01. ANOVA test was used to analyze statistical significance between groups. ANOVA, analysis of variance; OVA, ovalbumin.

We observed that ILC2s in PBMCs and BALF of asthmatic mice gated as the CD45^+^Lin^−^CD127^+^ST2^+^ population (Figure 5A,C). Moreover, intranasal IL‐33 administration to mice increased the ILC2 count in PBMCs and BALF. In contrast, anti‐IL‐33 antibody significantly decreased the number of ILC2s in PBMCs and BALF of mice with EA (Figure 5B,D). The secretion of IL‐5 and IL‐13 is essential for the effector functions of ILC2s. Accordingly, we noticed that IL‐33 significantly increased the number of activated IL‐5+ILC2s and IL‐13+ILC2s in mice with EA. Conversely, the numbers of IL‐5+ILC2s and IL‐13+ILC2s were significantly decreased in mice with EA after treatment with the anti‐IL‐33 antibody (Figure S6 A–F). Following stimulation with IL‐33, the expression of *Gata3* and *Rorα* in lung tissue was significantly increased, whereas the administration of anti‐IL‐33 antibody led to a decrease in their expression (Figure S7 A, B). In addition, we detected that mRNA expression of ILC2 transcription factors, including Crth2, Klrg1, and Icos, increased in the lung tissue of the OVA+IL‐33 group compared with that in other groups (Figure S7 C–E). Of note, the expression level of IL‐33 and ST2 mRNAs was higher in the OVA+IL‐33 group, whereas the administration of anti‐IL‐33 antibody decreased their expression in mice with EA (Figure S7 F–G). Consistently, IL‐33 increased the number of eosinophils in BALF, whereas anti‐IL‐33 antibody significantly decreased the number of eosinophils in mice with EA. Moreover, the number of neutrophils were reduced by IL‐33 treatment compared with that by the anti‐IL‐33 antibody in mice with EA (Figure S8 A–D). Histopathological examination of the lungs revealed that the OVA+IL‐33 group had the highest inflammation scores among the four groups of EA mice (Figure 9A, B, *p* < 0.05).

### 3.5 PI3K/AKT signaling pathway was involved in IL33/ILC2s

**FIGURE 6:**
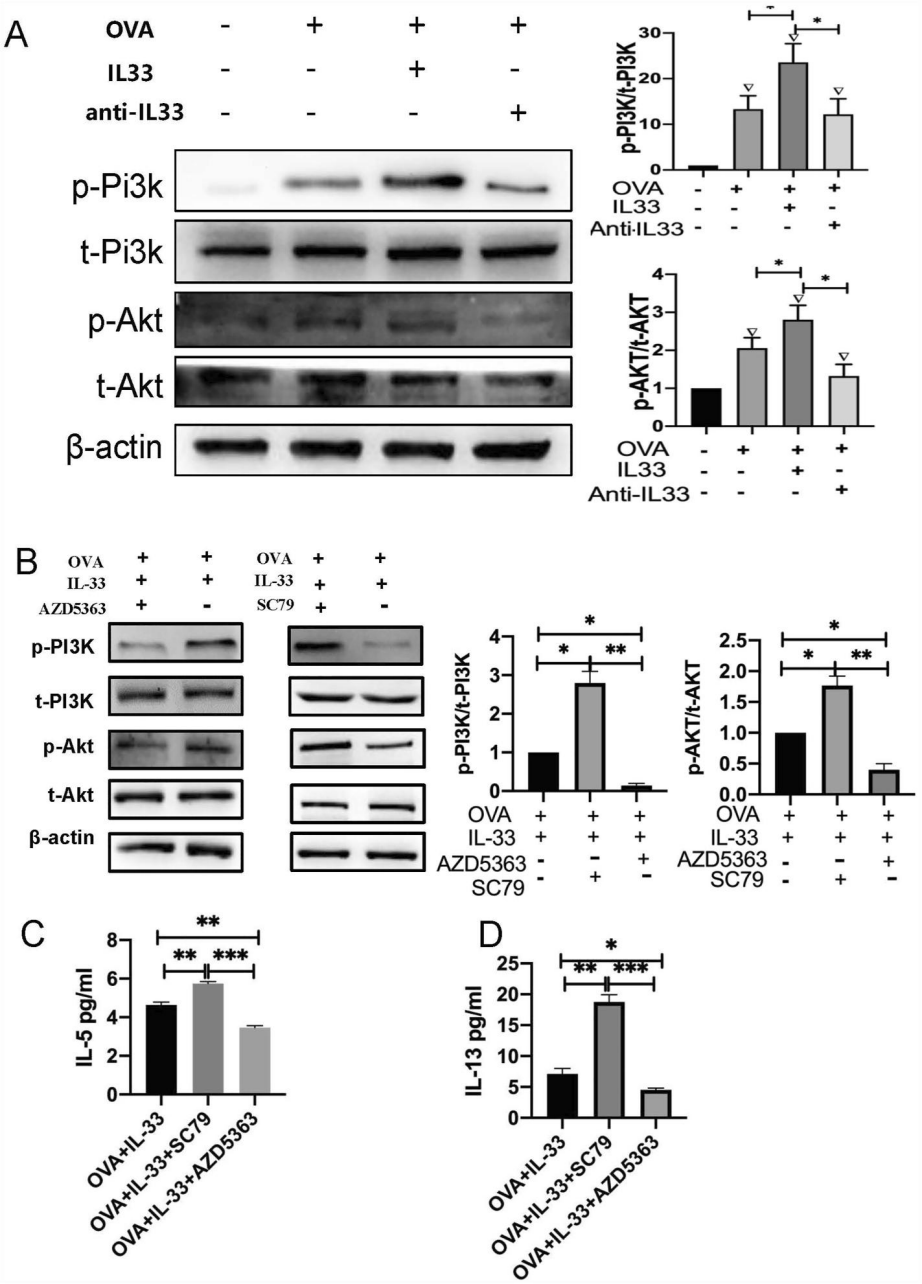
The p‐PI3K and p‐AKT signaling pathways were involved in IL33/innate lymphoid cells (ILC2s). (A) Expression of p‐PI3K, t‐PI3K, p‐AKT, and t‐AKT in isolated ILC2s as determined by performing western blot analysis (WB) (*n* = 4 mice). IL‐33 treatment increased the ratios of p‐PI3K/t‐PI3K and p‐AKT/t‐AKT, whereas the administration of anti‐Il‐33 antibody decreased these ratios in eosinophilic asthma (EA) mice. (B) The expression of p‐PI3K, t‐PI3K, p‐AKT, and t‐AKT in isolated ILC2s following treatment with an AKT inhibitor (AZD5363) or AKT agonist (SC79). (C–D) The production of IL‐5 and IL‐13 in isolated ILC2s following treatment with an AKT inhibitor (AZD5363) or AKT agonist (SC79). NC, negative control; p‐PI3K, phosphorylated PI3K; t‐PI3K, total PI3K; p‐AKT, phosphorylated AKT; t‐AKT, total AKT; AZD5363, AKT inhibitor; SC79, AKT agonist. ▽Comparison with NC group, ▽*p <* 0.05; *Comparison between different stimulated groups, *p < 0.05, **p < 0.01,***p < 0.001. ANOVA test was used to analyze statistical significance between groups. ANOVA, analysis of variance.

We isolated ILC2s from lung tissues to determine the effects of IL‐33 on the PI3K/AKT signaling pathway. We found that IL‐33 increased the ratio of p‐PI3K/t‐PI3K and p‐AKT/t‐AKT, whereas treatment with anti‐IL‐33 antibody decreased the ratio of p‐PI3K/p‐AKT in ILC2s obtained from EA mice (Figure 6A). In addition, the administration of the AKT inhibitor AZD5363 reduced the expression of p‐PI3K and p‐AKT. Conversely, treatment with the AKT agonist SC79 increased the expression of p‐PI3K and p‐AKT (Figure 6B). Furthermore, the AKT inhibitor AZD5363 reduced the levels of IL‐5 and IL‐13; in contrast, the AKT agonist SC79 significantly reversed the elevated levels of IL‐5 and IL‐13 (Figure 6C–D).

## 4 DISCUSSION

This study suggests that the number of ILC2s, especially those of activated ILC2s, and the associated gene expression of ILC2s are significantly elevated in EA. Hence, the numbers of ILC2s, which positively correlated with the count of blood eosinophils in our study, may be used to predict EA. Interestingly, IL‐33 induced the proliferation and pulmonary accumulation of ILC2s in EA. In addition, IL‐33 rapidly activated ILC2s and aggravated lung allergic inflammation in EA mice. Finally, IL‐33 upregulated the expression of p‐PI3K and p‐AKT proteins, affecting ILC2s.

Increased numbers of blood and sputum ILC2s are risk factors for severe asthma.^8^ Consistently, in our study, the numbers of ILC2s were elevated in patients with asthma, particularly in the EA group. The functions of ILC2s have already been investigated; the total number of IL‐5^+^ILC2s, IL‐13^+^ ILC2s, and CRTH2^+^ILC2s in the sputum significantly increased 24 h after allergen stimulation.^20^ Our results also revealed a significant increase in the counts of IL‐5^+^ILC2s and IL‐13^+^ILC2s in the blood and airways of mice with EA. Both GATA3 and RORa are necessary for the differentiation and maintenance of ILC2s.^21^ In addition, CRTH2 facilitates the chemotaxis of type 2 related immune cells and enhances the production of cytokines in vitro.^22^, ^23^ We observed substantially elevated mRNA expression levels of *GATA3* and *RORa* in EA, indicating a more active differentiation and proliferation of ILC2s. The relative mRNA expression of *CRTH2* was also increased, which stimulated ILC2s to produce type 2 cytokines in EA. In mice, ICOS signaling modulates ILC2s balance independently from T‐ and B‐cells by facilitating the proliferation and aggregation of pulmonary and intestinal ILC2s.^24^ Our study revealed that *ICOS* mRNA expression was increased in EA, which enhanced the proliferation and accumulation of mature lung ILC2s. *IL‐33* also activates ILC2s in type 2 immunity.^25^ Our study indicated that the increased mRNA expression of *IL‐*33 is due to the increased activation of ILC2s in EA.

In our study, the eosinophil count positively correlated with ILC2 number, but negatively correlated with the ACT. We then explored the diagnostic potential of ILC2s in eosinophilic airway inflammation. The number of eosinophils in IS contributes significantly to the diagnosis of eosinophilic airway inflammation. Nonetheless, patients with asthma tend to produce less sputum, making this procedure less feasible. Therefore, more readily available indicators are being explored for predicting eosinophilic airway inflammation. Our study revealed that the number of eosinophils in the blood can predict eosinophilic airway inflammation with high specificity; however, the detection sensitivity of eosinophils in blood was only 50%. Additionally, we confirmed that the levels of FeNO can predict eosinophilic airway inflammation. This process is easy to perform and does not cause discomfort in most patients. However, the levels of FeNO do not always reflect eosinophilic inflammation accurately.^26^ Therefore, ILC2 numbers in human blood may predict eosinophilic airway inflammation in patients with asthma with superior sensitivity, which would compensate for the lower sensitivity of blood eosinophils.

Our study demonstrated the proliferative capacity of ILC2s in EA. The in vitro proliferation of ILC2 was the strongest in the EA group treated with IL‐33. To observe the distribution of ILC2s in lung tissue following IL‐33 stimulation, we examined the localization of cells in lung tissue. The lack of specific ILC2 markers has limited our understanding of the in situ spatial organization of ILC2s. Additionally, ILC2s represent a small proportion of resident or infiltrating inflammatory cells, further limiting their in situ identification. For instance, ILC2s have not been reported in the lungs of wild mice.^27^ In this study, we used confocal microscopy to investigate the localization of ILC2s (GATA3+ CD3‐ICOS+) in wild‐type mice and found that ILC2s localized around blood vessels in the lungs. Moreover, we detected an increased number of ILC2s around blood vessels in the lungs of EA mice treated with IL‐33. These results suggest that IL‐33 might mediate the accumulation of activated ILC2s in the lungs.

IL‐33 activates ILC2s to produce IL‐5 and IL‐13, resulting in type‐2 innate immune response and allergic airway inflammation at mucosal barrier sites.^28^, ^29^ Our study showed that IL‐33 enhanced the numbers of IL‐5+ILC2s and IL‐13+ILC2s in EA mice, suggesting that IL‐33 not only affects the numbers of ILC2s but also activates them. We also explored the effect of IL‐33 on ILC2s at the genetic level. Increased expression of *Gata3* and *Rorα* mRNA following IL‐33 stimulation suggested that IL‐33 increased the differentiation and maturation of ILC2s in EA. In addition, IL‐33 induced *Crth2* and *Icos* mRNA expression, whereas ILC2 activation was significantly decreased after anti‐IL‐33 antibody administration. IL‐33 binds to its specific receptor ST2, which stimulates the production of IL‐13 and IL‐5.^30^ When tissues or cells are damaged, IL‐33 is released into the bloodstream. Subsequently, IL‐33 binds to ST2 on the surface of ILC2s, leading to the increased expression of *Gata3* and *Rorα* mRNA and stimulating ILC2 proliferation. Upon the intranasal administration of IL‐33, ILC2s accumulate in the lungs and BALF, causing severe asthma.^31^ Using IL‐33 receptor‐deficient mice or treating mice with soluble IL‐33 receptor or anti‐IL‐33 antibody, allergic inflammation was alleviated in patients with asthma.^32^ In our clinical and animal experiments, we found that an increased number of ILC2s was involved in the IL‐33‐mediated exacerbation of allergen‐induced asthmatic response and aggravated lung tissue inflammation; however, after treatment with anti‐IL‐33 antibody, both the numbers of ILC2s and lung tissue inflammation decreased in EA.

The mechanism underlying the contribution of IL‐33 and ILC2s to asthma exacerbation has not yet been established in patients with EA. The p38 MAPK, NF‐κB, PI3K/Akt, and Wnt/catenin pathways reportedly regulate IL‐33 transcription in endothelial cells in mice and humans.^14^ In patients with eosinophilic nasal polyps, IL‐33 induces the production of IL‐4 and IL‐5 via the PI3K/AKT pathway.^33^ Accordingly, PI3Kδ reduced the expression of IL‐33 and number of ILC2s, thereby inhibiting the allergic inflammatory response.^34^ Leptin, a small proline protein, and LAIR‐1 regulate ILC2s by targeting the PI3K‐AKT pathway, hence affecting the severity of asthma.^35^, ^36^, ^37^ Therefore, we hypothesized that IL‐33 and ILC2s play a role in asthma via the PI3K/AKT signaling pathway. In our study, IL‐33 upregulated the expression of PI3K and AKT proteins in ILC2s through ST2, increasing the levels of IL‐5 and IL‐13 (Th2 cytokines) and aggravating lung inflammation in patients with EA. Therefore, we propose that the PI3K/AKT pathway plays a mediating role in IL33/ILC2 and affects the subsequent inflammatory response.

Our study had certain limitations. We acknowledge that single‐center enrollment can lead to sample bias. This was mainly an observational study, and as such, our results on the mechanism of ILC2s and asthma need further validation. Our study demonstrated the impact of the PI3K/AKT signaling pathway on IL33/ILC2s. However, signaling pathways that regulate IL33/ILC2, such as the MAPK pathway, cannot be ruled out. Further studies should verify the role of the PI3K/AKT signaling pathway in IL33/ILC2s using various experimental models.

In summary, the numbers of ILC2s, especially those of activated ILC2s, were significantly higher in EA. Innate lymphoid cells from EA exhibited stronger proliferation in vitro, with IL‐33 inducing the pulmonary accumulation and rapid activation of ILC2s, consequently aggravating lung allergic inflammation. IL‐33 activated the expression of PI3K/AKT proteins, which are involved in the activation of ILC2s that might be important in aggravating eosinophilic inflammation in patients with EA. The findings of this study will guide further developments in the future diagnosis and assessment of EA.

## AUTHOR CONTRIBUTIONS

**Jing Liu**: Conceptualization, Visualization, Project administration, Supervision. **Fengfei Sun**: Conceptualization, Methodology, Writing‐Original Draft, Writing–Review and Editing. **Wei Zou, Honglei Shi**: Methodology, Software, Formal analysis. **Zehu Chen, Donghai Ma and Minmin Lin**: Methodology and data curation. **Kongqiu Wang, Yiying Huang**: Resources, Methodology. **Xiaobin Zheng**: Supervision, Resources. **Cuiyan Tan, Meizhu Chen, Changli Tu**: Resources, Data curation. **Zhenguo Wang, Jian Wu, Weiming Wu**: Resources.

## CONFLICT OF INTEREST STATEMENT

None declared.

## CONFERENCE PRESENTATION

None.

## Supporting information

Supplementary Material

Supplementary Material

Supplementary Material

Supplementary Material

Supplementary Material

Supplementary Material

Supplementary Material

Supplementary Material

Supplementary Material

Supplementary Material

Supplementary Material

## References

[1] Xu I , Boulay ME , Bertrand M , Côté A , Boulet LP . Comparative features of eosinophilic and non‐eosinophilic asthma. Clin Exp Allergy. 2022;52(1):205‐208. 10.1111/cea.13959 34053138

[2] Falquet M , Ercolano G , Jandus P , Jandus C , Trabanelli S . Healthy and patient type 2 innate lymphoid cells are differently affected by in vitro culture conditions. J Asthma Allergy. 2021;14:773‐783. 10.2147/jaa.s304126 34239308 PMC8259735

[3] Yasuda Y , Nagano T , Kobayashi K , Nishimura Y . Group 2 innate lymphoid cells and the house dust mite‐induced asthma mouse model. Cells. 2020;9(5):1178. 10.3390/cells9051178 32397396 PMC7290734

[4] Arae K , Ikutani M , Horiguchi K , et al. Interleukin‐33 and thymic stromal lymphopoietin, but not interleukin‐25, are crucial for development of airway eosinophilia induced by chitin. Sci Rep. 2021;11(1):5913. 10.1038/s41598-021-85277-4 33723298 PMC7960735

[5] Bønnelykke K , Sleiman P , Nielsen K , et al. A genome‐wide association study identifies CDHR3 as a susceptibility locus for early childhood asthma with severe exacerbations. Nat Genet. 2014;46(1):51‐55. 10.1038/ng.2830 24241537

[6] Moffatt MF , Gut IG , Demenais F , et al. A large‐scale, consortium‐based genomewide association study of asthma. N Engl J Med. 2010;363(13):1211‐1221. 10.1056/nejmoa0906312 20860503 PMC4260321

[7] Gudbjartsson DF , Bjornsdottir US , Halapi E , et al. Sequence variants affecting eosinophil numbers associate with asthma and myocardial infarction. Nat Genet. 2009;41(3):342‐347. 10.1038/ng.323 19198610

[8] Bartemes KR , Kephart GM , Fox SJ , Kita H . Enhanced innate type 2 immune response in peripheral blood from patients with asthma. J Allergy Clin Immunol. 2014;134(3):671‐678.e4. 10.1016/j.jaci.2014.06.024 25171868 PMC4149890

[9] Chen R , Smith SG , Salter B , et al. Allergen‐induced increases in sputum levels of group 2 innate lymphoid cells in subjects with asthma. Am J Respir Crit Care Med. 2017;196(6):700‐712. 10.1164/rccm.201612-2427oc 28422515

[10] Busse WW , Gern JE . Weaving innate lymphoid cells (ILCs) into the fabric of asthma exacerbations. J Allergy Clin Immunol. 2022;149(5):1579‐1581. 10.1016/j.jaci.2022.01.021 35149042

[11] Dhariwal J , Cameron A , Wong E , et al. Pulmonary innate lymphoid cell responses during rhinovirus‐induced asthma exacerbations in vivo: a clinical trial. Am J Respir Crit Care Med. 2021;204(11):1259‐1273. 10.1164/rccm.202010-3754oc 34469272 PMC8786078

[12] Liu T , Wu J , Zhao J , et al. Type 2 innate lymphoid cells: a novel biomarker of eosinophilic airway inflammation in patients with mild to moderate asthma. Respir Med. 2015;109(11):1391‐1396. 10.1016/j.rmed.2015.09.016 26459159

[13] Li Y , Fu Y , Chen H , Liu X , Li M . Blocking interleukin‐33 alleviates the joint inflammation and inhibits the development of collagen‐induced arthritis in mice. J Immunol Res. 2020;2020:1‐8. 10.1155/2020/4297354 PMC7801941 33490289

[14] Duez C , Gross B , Marquillies P , Ledroit V , Ryffel B , Glineur C . Regulation of IL (Interleukin)‐33 production in endothelial cells via kinase activation and Fas/CD95 upregulation. Arterioscler Thromb Vasc Biol. 2020;40(11):2619‐2631. 10.1161/atvbaha.120.314832 32907372

[15] Stark AK , Davenport ECM , Patton DT , et al. Loss of phosphatidylinositol 3‐kinase activity in regulatory T cells leads to neuronal inflammation. J Immunol. 2020;205(1):78‐89. 10.4049/jimmunol.2000043 32414808 PMC7311201

[16] Sun F , Liang Y , Lin M , et al. Regulatory T cell deficiency in patients with eosinophilic asthma. J Asthma. 2022;59(9):1703‐1711. 10.1080/02770903.2021.1962908 34346277

[17] Hayashi T , Adachi Y , Hasegawa K , Morimoto M . Less sensitivity for late airway inflammation in males than females in BALB/c mice. Scand J Immunol. 2003;57(6):562‐567. 10.1046/j.1365-3083.2003.01269.x 12791094

[18] Jiang M , Liu H , Li Z , et al. ILC2s induce adaptive Th2‐type immunity in acute exacerbation of chronic obstructive pulmonary disease. Mediat Inflamm. 2019;2019:3140183. 10.1155/2019/3140183 PMC6610743 31320835

[19] Mjosberg JM , Trifari S , Crellin NK , et al. Human IL‐25‐ and IL‐33‐responsive type 2 innate lymphoid cells are defined by expression of CRTH2 and CD161. Nat Immunol. 2011;12(11):1055‐1062. 10.1038/ni.2104 21909091

[20] Roberts LB , Schnoeller C , Berkachy R , et al. Acetylcholine production by group 2 innate lymphoid cells promotes mucosal immunity to helminths. Sci Immunol. 2021;6(57):eabd0359. 10.1126/sciimmunol.abd0359 33674321

[21] Hoyler T , Klose CS , Souabni A , et al. The transcription factor GATA‐3 controls cell fate and maintenance of type 2 innate lymphoid cells. Immunity. 2012;37(4):634‐648. 10.1016/j.immuni.2012.06.020 23063333 PMC3662874

[22] Chang JE , Doherty TA , Baum R , Broide D . Prostaglandin D2 regulates human type 2 innate lymphoid cell chemotaxis. J Allergy Clin Immunol. 2014;133(3):899‐901.e3. 10.1016/j.jaci.2013.09.020 24210841 PMC3943597

[23] Wojno ED , Monticelli LA , Tran SV , et al. The prostaglandin D_₂_ receptor CRTH2 regulates accumulation of group 2 innate lymphoid cells in the inflamed lung. Mucosal Immunol. 2015;8(6):1313‐1323. 25850654 10.1038/mi.2015.21 PMC4598246

[24] Paclik D , Stehle C , Lahmann A , Hutloff A , Romagnani C . ICOS regulates the pool of group 2 innate lymphoid cells under homeostatic and inflammatory conditions in mice. Eur J Immunol. 2015;45(10):2766‐2772. 10.1002/eji.201545635 26249010

[25] Kondo Y , Yoshimoto T , Yasuda K , et al. Administration of IL‐33 induces airway hyperresponsiveness and goblet cell hyperplasia in the lungs in the absence of adaptive immune system. Int Immunol. 2008;20(6):791‐800. 10.1093/intimm/dxn037 18448455

[26] Nair P , Kjarsgaard M , Armstrong S , Efthimiadis A , O'Byrne PM , Hargreave FE . Nitric oxide in exhaled breath is poorly correlated to sputum eosinophils in patients with prednisone‐dependent asthma. J Allergy Clin Immunol. 2010;126(2):404e406‐406. 10.1016/j.jaci.2010.05.032 20621343

[27] Puttur F , Denney L , Gregory LG , et al. Pulmonary environmental cues drive group 2 innate lymphoid cell dynamics in mice and humans. Sci Immunol. 2019;4(36):eaav7638. 10.1126/sciimmunol.aav7638 31175176 PMC6744282

[28] Barlow JL , Peel S , Fox J , et al. IL‐33 is more potent than IL‐25 in provoking IL‐13‐producing nuocytes (type 2 innate lymphoid cells) and airway contraction. J Allergy Clin Immunol. 2013;132(4):933‐941. 10.1016/j.jaci.2013.05.012 23810766

[29] Martinez‐Gonzalez I , Matha L , Steer CA , Ghaedi M , Poon G , Takei F . Allergen‐experienced group 2 innate lymphoid cells acquire memory‐like properties and enhance allergic lung inflammation. Immunity. 2016;45(1):198‐208. 10.1016/j.immuni.2016.06.017 27421705

[30] Vivier E , Artis D , Colonna M , et al. Innate lymphoid cells: 10 Years on. Cell. 2018;174(5):1054‐1066. 10.1016/j.cell.2018.07.017 30142344

[31] Klein Wolterink RG , Kleinjan A , van Nimwegen M , et al. Pulmonary innate lymphoid cells are major producers of IL‐5 and IL‐13 in murine models of allergic asthma. Eur J Immunol. 2012;42(5):1106‐1116. 10.1002/eji.201142018 22539286

[32] Sjoberg LC , Nilsson AZ , Lei Y , Gregory JA , Adner M , Nilsson GP . Interleukin 33 exacerbates antigen driven airway hyperresponsiveness, inflammation and remodeling in a mouse model of asthma. Sci Rep. 2017;7(1):4219. 10.1038/s41598-017-03674-0 28652606 PMC5484710

[33] Luo X , Li C , Wang Y , et al. Interleukin‐33 promotes Th2/Th17 response in eosinophilic and non‐eosinophilic nasal polyps. ORL J Otorhinolaryngol Relat Spec. 2020;82(1):34‐39. 10.1159/000503976 31778997

[34] Uddin S , Amour A , Lewis DJ , et al. PI3Kδ inhibition prevents IL33, ILC2s and inflammatory eosinophils in persistent airway inflammation. BMC Immunol. 2021;22(1):78. 10.1186/s12865-021-00461-5 34920698 PMC8684271

[35] Helou DG , Shafiei‐Jahani P , Hurrell BP , et al. LAIR‐1 acts as an immune checkpoint on activated ILC2s and regulates the induction of airway hyperreactivity. J Allergy Clin Immunol. 2022;149(1):223‐236.e6. 10.1016/j.jaci.2021.05.042 34144112 PMC8674385

[36] Zeng Q , Luo X , Tang Y , Liu W , Luo R . Leptin regulated ILC2 cell through the PI3K/AKT pathway in allergic rhinitis. Mediat Inflamm. 2020;2020:4176082‐4176089. 10.1155/2020/4176082 PMC7081033 32214904

[37] Zhu G , Cai H , Ye L , et al. Small proline‐rich protein 3 regulates IL‐33/ILC2 Axis to promote allergic airway inflammation. Front Immunol. 2021;12:758829. 10.3389/fimmu.2021.758829 35126350 PMC8810634

