# Supplementary material for: Interleukin‐33 increases type 2 innate lymphoid cell count and their activation in eosinophilic asthma
Source: Clin Transl Allergy. 2023 Jun 1;13(6):e12265. doi: 10.1002/clt2.12265 (PMC10234174; doi:10.1002/clt2.12265)
Supplement: Supplementary file 1 — Supplementary Material [file CLT2-13-e12265-s009.docx]

**IL-33 increases type 2 innate lymphoid cell count and their activation in eosinophilic asthma**

**Supplementary material**

**Methods**

**Inclusion/exclusion criteria for selecting study subjects**

The inclusion criteria for patients with asthma included diagnosis according to the Global Strategy for Asthma Management and Prevention (GINA): the diagnosis of asthma was made by experienced pulmonologists and was based on the history of asthma symptoms, variable airflow limitation, plus one of the following objectives tests: 1) Positive bronchodilator responsiveness: increase inFEV1 of >12% and >200 ml from baseline level ten minutes after 200 mcg of salbutamol; 2) Positive bronchial challenge test: decrease in FEV1 from baseline of ≥20% with standard doses of methacholine; Volunteering to participate in this study and signing the informed consent; Age ≥ 18 years, patients of either sex; No serious diseases of the heart, lungs, brain, liver, kidneys, and blood system, or other diseases; life expectancy of not less than 1 year. Subjects were excluded if they were diagnosed with acute viral infections within at least 1 month prior to the study, or with chronic obstructive pulmonary disease, cardiovascular disease, immunodeficiency, and cystic fibrosis, or if they were pregnant. The inclusion criteria for HC were age over 18 years; No history of immune or allergic diseases; Volunteering to participate in the study and signing the consent form; And no use of corticosteroids or other immunosuppressive drugs in 4 weeks prior to study commencement.

**Murine model of eosinophilic asthma**

Ovalbumin was used for sensitization and 1% OVA aerosol in saline was used to induce asthma (through inhalation). For generating the EA model, mice were peritoneally immunized using 20 μg OVA emulsified in 100 μL alum (2.25 mg aluminum hydroxide) on day 0 and 14, followed by ultrasonic nebulization with 1% OVA aerosol (40 min) on days 24, 25, and 26. Increased eosinophil but not neutrophils in lung tissue or bronchoalveolar lavage fluid and in blood were shown in mouse model of asthma, which was defined as EA model of mice^1^. In the OVA+IL33 group, rIL33 (0.5 μg intranasally (i.n.)) once every other day, three times in total) was administered after forced inhalation of 1% OVA aerosol for 30 min; in OVA+anti-IL33 group, mice were nebulized with 1% OVA aerosol for 30 min and then treated with the anti-IL33 antibody (50 mg i.p. for six consecutive days). Mice in the control group were treated with an equivalent volume of PBS.

**Induced sputum and classification of inflammatory cells**

Sputum specimens were obtained as previously reported.^2^ Sputum was filtered and tested for cell viability and total cell count. Sputum samples containing less than 80% squamous cells were included in analysis. Individuals were categorized according to the proportion of neutrophils and eosinophils in the sputum. Individuals with ≥3 % eosinophils in sputum were considered having eosinophilic asthma (EA), whereas those with ≥61 % neutrophils were considered neutrophilic asthma (NA). Paucigranulocytic asthma (PA) was characterized by eosinophil < 3% and neutrophil < 61%. Patients with asthma were classified as mixed asthma (MA) if they had ≥ 3% eosinophils and ≥ 61% neutrophils. EA defined as eosinophils ≥3% in sputum. NEA included these kinds of asthma (NA, PA and MA).

**Cell staining and inflammatory cell count**

The severity of lesions in each transversely sectioned bronchus-bronchiole was evaluated by light microscopy and scored as follows: no lesions, 0; mucus or desquamated epithelial cells in the lumen, 1; a few goblet cells in the epithelial layer, 1; many such cells, 2; thickened basement membrane, 1; mild infiltration of eosinophils and lymphocytes in the mucosa, 1; severe infiltration in the mucosa, 2; hypertrophy of smooth muscle cells in mucosa, 1. For each mouse, the scoring index (SI) was computed by dividing the total score by the number of inspected bronchioles, and the mean SI of each group was ascertained blindly (n = 10/group) by two independent investigators.

**Evaluation of ILC2-related gene expression using RT-qPCR**

Total RNA from human peripheral blood or mouse lung tissues was extracted using TRIzol LS (Thermo Fisher, Cal,USA) and reverse transcribed using cDNA synthesis kits (Thermo Fisher, Cal,USA). This was followed by real-time PCR using SYBR™ Master Mix (Thermo Fisher, Cal,USA) on a CFX96 detection system (Bio-Rad, Cal, USA) according to the manufacturer’s instructions. Relative expression of genes encoding GATA3, RORa, CD127, CRTH2, ICOS, KLRG1, and IL33 was determined using the 2−ΔΔCq method^3^ and standardized to expression of gene encoding GAPDH.

**Primer sequences**

Primer sequences used in the study are as follows:

Human primer sequences

| Gene | primer sequences (5′-3′) |
| --- | --- |
| *GATA3* | Forward：GCCCCTCATTAAGCCCAAG  Reverse：TTGTGGTGGTCTGACAGTTCG |
| *RORa* | Forward：CTTGCCGTAGGGATGTCTCG  Reverse：GAAGTTCCGTCAGCCCGTT |
| *CD127* | Forward：CCCTCGTGGAGGTAAAGTGC  Reverse：CCTTCCCGATAGACGACACTC |
| *CRTH2* | Forward：TCTGCACTTCCAACTTAGGCG  Reverse：GCTCTGGAGACGGCTCATC |
| *ICOS* | Forward：CAGGAGAAATCAATGGTTCTGCC  Reverse：CGCATTTTTAACTGCTGGACAG |
| *KLRG1* | Forward：TCCATGTTAGAGTTGCCTACGG  Reverse：AAGTGGAGTAGTTGAGCCCT |
| *IL33* | Forward：GTGACGGTGTTGATGGTAAGAT  Reverse：AGCTCCACAGAGTGTTCCTTG |
| *GAPDH* | Forward：TGTGGGCATCAATGGATTTGG  Reverse：ACACCATGTATTCCGGGTCAAT |

Mice primer sequences

| Gene | primer sequences (5′-3′) |
| --- | --- |
| *Gata3* | Forward：CTCGGCCATTCGTACATGGAA  Reverse：GGATACCTCTGCACCGTAGC |
| *Rora* | Forward：GTGGAGACAAATCGTCAGGAAT  Reverse：TGGTCCGATCAATCAAACAGTTC |
| *Cd127* | Forward：GCGGACGATCACTCCTTCTG  Reverse：AGCCCCACATATTTGAAATTCCA |
| *Crth2* | Forward：AGATGGTCCAGCTTCCAAACC  Reverse：ACAGGATGAGTCCGTTTTCCA |
| *Icos* | Forward：ATGAAGCCGTACTTCTGCCG  Reverse：CGCATTTTTAACTGCTGGACAG |
| *Klrg1* | Forward：TTTGGGGCTTTTGACTGTGAT  Reverse：TGTAAGGAGATGTGAGCCTTTGT |
| *IL-33* | Forward：ATTTCCCCGGCAAAGTTCAG  Reverse：AACGGAGTCTCATGCAGTAGA |
| *Gapdh* | Forward：CCTGCACCACCAACTGCTTA  Reverse：GGCCATCCACAGTCTTCTGAG |

**Sorting ILC2s in lung tissues of mice with EA**

Lung tissues extracted from five mice with EA were stained with FITC-conjugated lineage-positive (Lin^+^) antibodies against CD2, CD3, CD14, CD16, CD19, CD56, and CD235a, and FITC-conjugated anti-FceR1 antibody. Lin^-^ cells were subjected to negative selection using the EasySep FITC selection kit (StemCell Technologies, Vancouver, BC, Canada) according to the manufacturer’s instructions. Isolated Lin- cells were stained with PE-conjugated anti-CRTH2(BioLegend, San Diego, CA, USA) and PE-Cy7-conjugated anti-CD127 antibodies (BioLegend, San Diego, CA, USA) and then sorted into three populations based on the expression of CRTH2 and CD127 (Lin- CD127+ST2+) using FACSAria (Beckman, BD, USA). The final isolated fraction contained ILC2s that were up to 94% purified, as determined by flow cytometry. Sorted ILC2s were then cultured in vitro.

**In vitro proliferation capacity of ILC2s**

The sorted ILC2s were cultured according to previous methods,^12^ then divided into: the HC/NC group: ILC2s obtained from HC/NC+IL-2+IL-25; HC/NC+IL-33 group: ILC2s obtained from HC/NC +IL-2+IL-25+IL-33; EA group : ILC2s obtained from EA + IL-2+IL-25; EA+IL-33 group : ILC2s obtained from EA + IL-2+IL-25 +IL-33. Briefly, 500 cells per well were grown in 96-well plates with incomplete Iscove's modified Dulbecco's medium containing 500 ng/mL IL-2 (PeproTech, New Jersey, USA), 500 ng/mL IL-25 (PeproTech, New Jersey, USA), and 500 ng/mL IL-33 (R&D) for 5 d. Subsequently, 10 μL CCK8 solution was added to each well, taking care not to generate air bubbles during this process. Plates were incubated at 37 °C for 1–4 h, and finally, a microplate reader was used to measure the absorbance at 450 nm. And then relative cell viability was calculated to represent proliferation of cells.

**Western blot analysis**

ILC2s were also exposed to the AKT agonist, SC79 (4 μg/mL; Selleck) for 24 h. Subsequently, ILC2s were lysed using RIPA lysis buffer (Solarbio, Beijing, China) containing 1 mM PMSF (Solarbio, Beijing, China). Total protein in the extract was quantified using a bicinchoninic acid (BCA) protein assay kit. Subsequently, equal amounts of total protein (20 μg) were subjected to sodium dodecyl sulfate-polyacrylamide gel electrophoresis (SDS-PAGE) and transferred to polyvinylidene fluoride (PVDF) membranes (Bio-Rad, Cal, USA). After blocking in 5% nonfat milk for 1 h at 25  °C, membranes were incubated with antibodies against PI3K (CST, Boston, MA, USA), p-PI3K (CST, Boston, MA, USA), AKT (CST, Boston, MA, USA), p-AKT (CST, Boston, MA, USA), and β-actin (Proteintech Group,Wuhai, China) overnight at 4 °C. Membranes were then incubated with horseradish peroxidase (HRP)-conjugated secondary antibodies. Images were acquired using an iBright 1500 imaging system (Thermo Fisher, Cal,,USA) using an ECL reagent (Solarbio, Beijing, China). Relative protein expression was calculated as the ratio of phosphorylated protein to total protein.

**Statistical analyses**

Multiple comparisons refer to the circumstance where there are 3 or more separate groups being compared on a given outcome variable. Firstly, data must be isolation. Then, test equal variance should be performed. If the test equal variance >0.05, the population variances will be considered equal. If the test equal variance <0.05, the population will not equal. We need correct it using these methods such as Welch corrections or Brown-Forsythe. Finally, according to the equal or uneven variance, we performed one-way ANOVA or Kruskal-Wallis test analysis to determine whether there was a difference between the population means. If the results are different, multiple comparisons with LSD could be used to further determine which of the two means was different. The variables involved of ROC analysis were based on statistically significant from the Spearman rank test.

REFERENCES

1. Kianmeher M, Ghorani V, Boskabady MH. Animal Model of Asthma, Various Methods and Measured Parameters: A Methodological Review.  *Iran J Allergy asthma Immunol.* 2016;15(6):445-465.
2. Sun F, Liang Y, Lin M, et al., Regulatory T cell deficiency in patients with eosinophilic asthma. *J Asthma*. 2022;59(9):1703–1711.
3. Analysis of relative gene expression data using real-time quantitative PCR and the 2-ΔΔCq method. Methods 25: 402-408, 2001.

**TABLES**

**Table S1.** Lung function of patients with asthma with different inflammatory phenotypes

|  | **EA** | | **NEA** | **P** |
| --- | --- | --- | --- | --- |
| FEV1(pre)%predict | | 69.37 (56.41/77.55) | 77.86 (66.13/82.91) | 0.0004 |
| FEV1/FVC | | 83.95 (67.45/94.25) | 93.6 (81.8/99.6) | 0.0015 |
| ^△^FEV1% (improvement) | | 12.79 (5.13/23.87) | 10.79 (4.69/15.36) | 0.049 |
| Z5 (% predicted) | | 216.2 (140.5/326.2) | 136.9 (105/171.7) | 0.0185 |
| Z5 (kPa/l/s) | | 0.635 (0.40/1.16) | 0.51 (0.43/0.62) | 0.32 |
| R5 (% predicted) | | 180.7 (137.9/271) | 133.4 (102.8/166.8) | 0.0206 |
| R5 (kPa/l/s) | | 0.57 (0.39/0.84) | 0.49 (0.41/0.59) | 0.27 |
| R20 (% predicted) | | 152.8 (130.0/189.3) | 130.9 (113.1/143.5) | 0.0316 |
| R20 (kPa/l/s) | | 0.39 (0.35/0.49) | 0.41 (0.38/0.46) | 0.41 |
| R5-R20 (% predicted) | | 24.65 (-21.35/100) | 0.75 (-11.83/15.35) | 0.05 |
| Fres (HZ) | | 73.62 (38.2/145.1) | 36 (23.07/47.55) | 0.035 |
| (ppb) 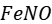 | | 51 (21/90) | 20 (15/33) | 0.0005 |

Data are presented as median (25–75 %) IQR. EA, eosinophilic asthma; NEA, non-eosinophilic asthma. Man-Whitney U-test was used to analyze statistical significance between groups.

**Table S2.** Relevant factors of ROC analysis for eosinophilic inflammation

|  | **AUC** | **P-value** | **95 % confidence interval**  **Lower** **upper** | | **Cut-off value** | **Sensitivity** | **Specificity** |
| --- | --- | --- | --- | --- | --- | --- | --- |
| **% ILC2s in blood** | 0.731 | 0.034 | 0.548 | 0.914 | 0.065 | 89 % | 50 % |
| **EOS in blood** | 0.708 | 0.05 | 0.517 | 0.9 | 0.465 | 50 % | 91.7 % |
| **Serum IgE** | 0.597 | 0.38 | 0.391 | 0.804 | 839.15 | 27.8 % | 100 % |
| **ACT** | 0.542 | 0.7 | 0.703 | 0.324 | 16.5 | 55.6 % | 50 % |
| **FeNO** | 0.741 | 0.028 | 0.554 | 0.927 | 39.5 | 66.7 % | 91.7 % |

AUC and p-values for separate variables are shown. AUC: area under the curve.

**FIGURE LEGENDS**

**Fig S1.** Flow chart showing the study groups.

**Fig S2.** The counts of IL-5^+^ILC2s and IL-13^+^ILC2s were elevated in EA.

1. IL-5+ILC2s and IL-13+ILC2s were gated as the IL-5+CD45+Lin-CD127+CD294+ and IL-13+CD45+Lin-CD127+CD294+ population in PBMCs obtained from the HC and patients with asthma. (B) The numbers of IL-5+ILC2s were significantly higher in PBMCs obtained from the EA group than those in HC and NEA groups. (C) The numbers of IL-13+ILC2s were significantly higher in PBMCs of the EA group than those in HC and NEA groups.

HC, healthy control; EA, eosinophilic asthma; NEA, non-eosinophilic asthma. ▽Comparison with HC group, ▽*P < 0.05;* *Comparison with EA group, **P < 0.05;* Kruskal-Wallis test was used to analyze statistical significance between groups.

**Fig S3.** The expression of *GATA3*, *RORa*, *CRTH2*, and *IL-33* was increased in EA.

(A-G) The expression of ILC2s-related mRNAs in PBMCs of HC and patients with asthma.

HC, healthy control; EA, eosinophilic asthma; NEA, non-eosinophilic asthma *GATA3,* GATA-binding protein 3; *RORa,* retinoid-related orphan receptor α; *CD127*: Interleukin-7 receptor alpha*; CRTH2,* chemoattractant receptor homologous molecule expressed on Th2 cells; ICOS, inducible CO-stimulator; KLRG1, killer cell lectin-like receptor G1; *IL-33:* Interleukin-33.▽Comparison with HC group, ▽*P < 0.05;* *Comparison with EA group, **P < 0.05, **P < 0.01;* Kruskal-Wallis test was used to analyze statistical significance between groups.

**Fig S4.** Patients with eosinophilic asthma had worse lung function.

(A-I) Comparison of lung function in four asthma phenotype groups.

EA: eosinophilic asthma, NEA: non-eosinophilic asthma, NA: neutrophilic asthma, MA: mixed asthma, PA: pauci-granulocytic asthma. *Comparison between difference groups, **P <* 0.05*, **P < 0.01, ***P < 0.001;* Kruskal-Wallis test was used to analyze statistical significance between groups.

**Fig S5.** ILC2s as a potential predictor of eosinophilic airway inflammation.

ROC analysis of ILC2s percentage in blood, eosinophils in blood, FeNO level, serum IgE level, and ACT for the diagnosis of eosinophilic airway inflammation in asthma. ROC: receiver operating characteristic; AUC: area under the curve.

**Fig S6.** IL-33 enhanced the proportion of IL-5+ILC2s and IL-13+ILC2s in PBMCs and BALF in EA mice.

1. IL-5^+^ILC2s and IL-13^+^ILC2s were gated as the IL-5^+^CD45^+^Lin^-^CD127^+^ST2^+^ and IL-13^+^CD45^+^Lin^-^CD127^+^ST2^+^ population in PBMCs obtained from mice. (B) IL-5+ILC2s and IL-13+ILC2s were gated as the IL-5+CD45+Lin-CD127+ST2+ and IL-13+CD45+Lin-CD127+ST2+ population in BALF obtained from mice. (C-D) The numbers of IL-5^+^ILC2s and IL-13^+^ILC2s in PBMCs were highest in the OVA+IL-33 group. (E-F) The numbers of IL-5+ILC2s and IL-13+ILC2s in BALF were significantly higher in the OVA+IL-33 group.

NC, negative control; EA, eosinophilic asthma; MC, mononuclear cell; BALF, bronchoalveolar lavage fluid. ▽Comparison with NC group, ▽*P < 0.05;* ∗Comparison with OVA group, *∗P < 0.05;* #Comparison with OVA+IL-33 group*; ##P < 0.01;* ANOVA test was used to analyze statistical significance between groups.

**Fig S7.** ILC2 transcription factors were upregulated in the OVA+IL-33 group.

(A-G) The expression of ILC2s-related mRNAs in lung tissues of mice in the four groups.

NC, negative control; EA, eosinophilic asthma; *Gata3,* GATA-binding protein 3; *Rora,* retinoid-related orphan receptor α; *Crth2,* chemoattractant receptor homologous molecule expressed on Th2 cells; Klrg1, killer cell lectin-like receptor G1; Icos, inducible CO-stimulator; *Il-33:* Interleukin-33; ST2, growth stimulation expressed gene 2. ▽Comparison with NC group, ▽*P < 0.05;* *Comparison with OVA group, **P < 0.05, **P < 0.01;* #Comparison with OVA+IL-33 group*; #P < 0.05, ##P < 0.01, ###P < 0.001;* ANOVA test was used to analyze statistical significance between groups.

**Fig S8**. The percentage of eosinophils in BALF and OVA-special IgE in serum were higher in the OVA+IL-33 group.

1. HE-stained eosinophils in BALF of the four groups. (B) The percentage of EOS in BALF. (C) The percentage of NEU in BALF. (D) Serum level of OVA-special IgE.

NC: negative control mice; EOS: eosinophils; NEU: neutrophils. ▽Comparison with NC group; ▽*P < 0.05;* ▽▽*P < 0.01;* ▽▽▽*P < 0.001;* *Comparison with OVA group*;* ***P < 0.01. #*Comparison with the OVA+IL-33 group*; ##P < 0.01;* ANOVA test was used to analyze statistical significance between groups.

**Fig S9.** IL-33 aggravated pulmonary inflammation in mice with EA

1. Histopathologic characteristics of HE-stained lung tissues in mice with EA. (B) Inflammation scores in lung tissues were highest in the OVA+IL-33 group.

NC: negative control. ▽Comparison with NC group; ▽*P < 0.05;* ▽▽▽*P < 0.001;* *Comparison with OVA group, ***P <* 0.01*;* ****P <* 0.001; #Comparison with OVA+IL-33 group*; ##P < 0.01; ###P < 0.001;* ANOVA test was used to analyze statistical significance between groups.
