# Supplementary figures and images for: Interleukin‐33 increases type 2 innate lymphoid cell count and their activation in eosinophilic asthma
Source: Clin Transl Allergy. 2023 Jun 1;13(6):e12265. doi: 10.1002/clt2.12265 (PMC10234174; doi:10.1002/clt2.12265)

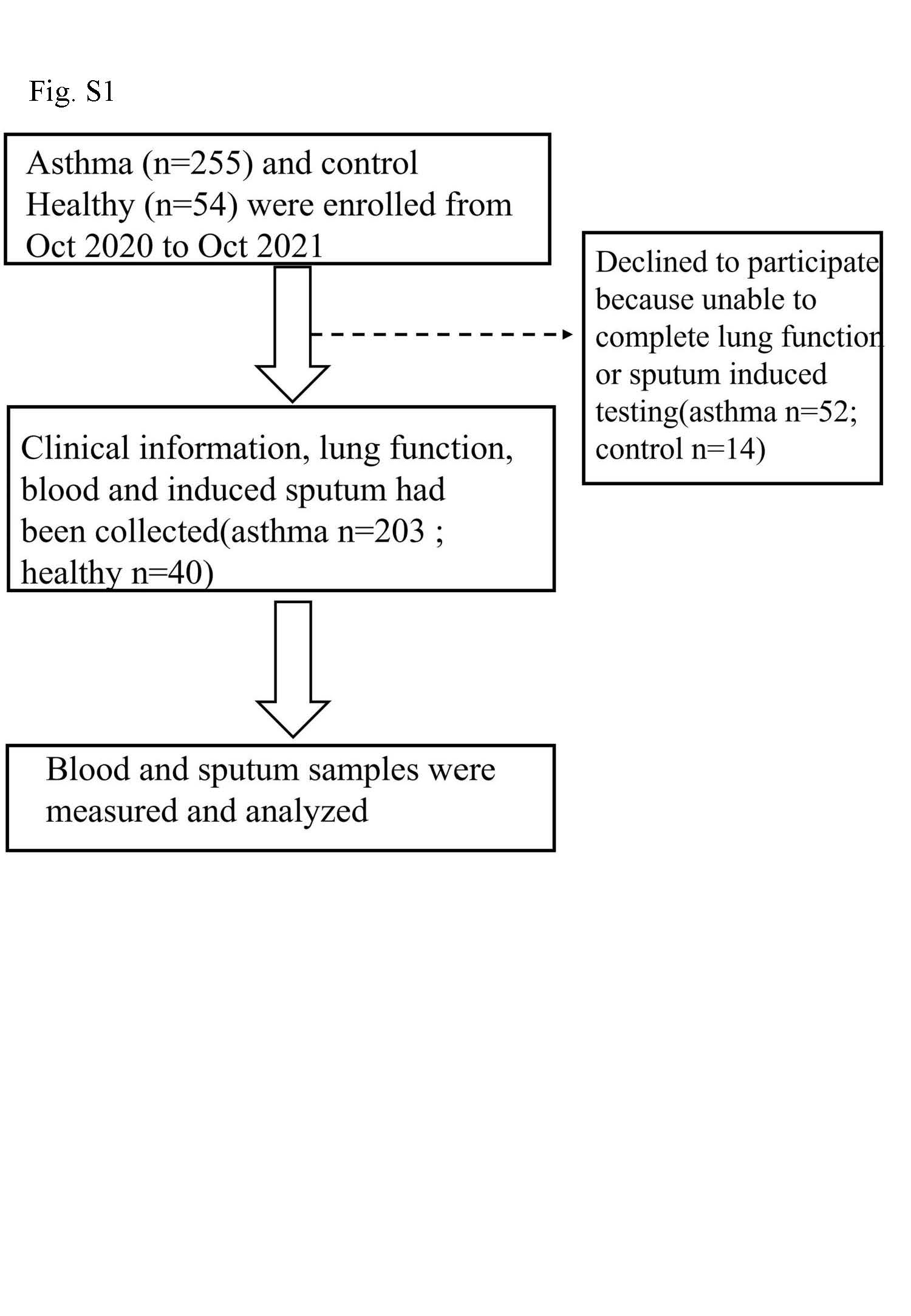

Supplement: Supplementary file 2 — Supplementary Material [file CLT2-13-e12265-s003.jpg]

## Slide 1
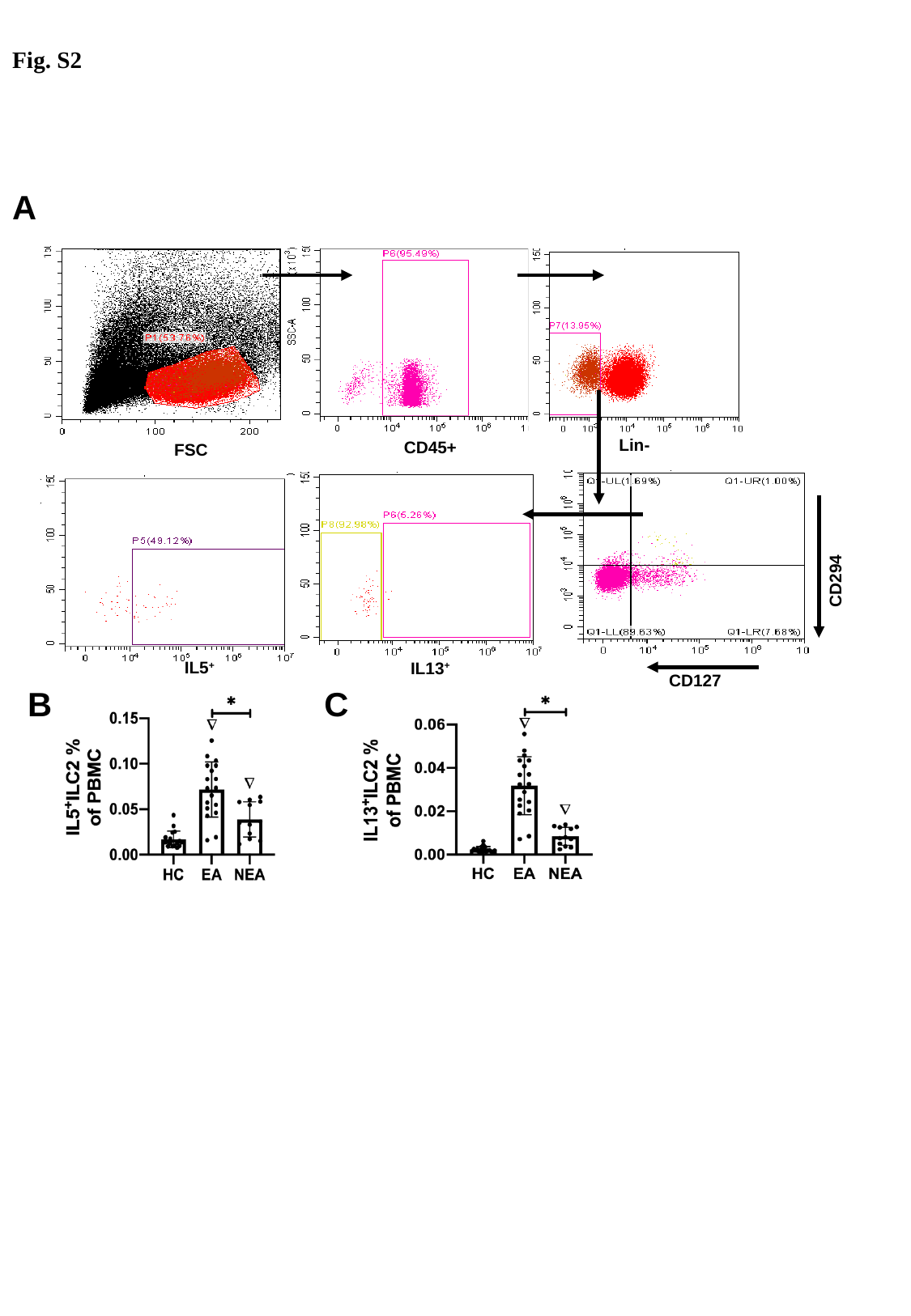

Fig. S2
A
FSC
CD45+
Lin-
CD294
CD127
IL13+
IL5+
B
C

Supplement: Supplementary file 3 — Supplementary Material [file CLT2-13-e12265-s004.pptx]

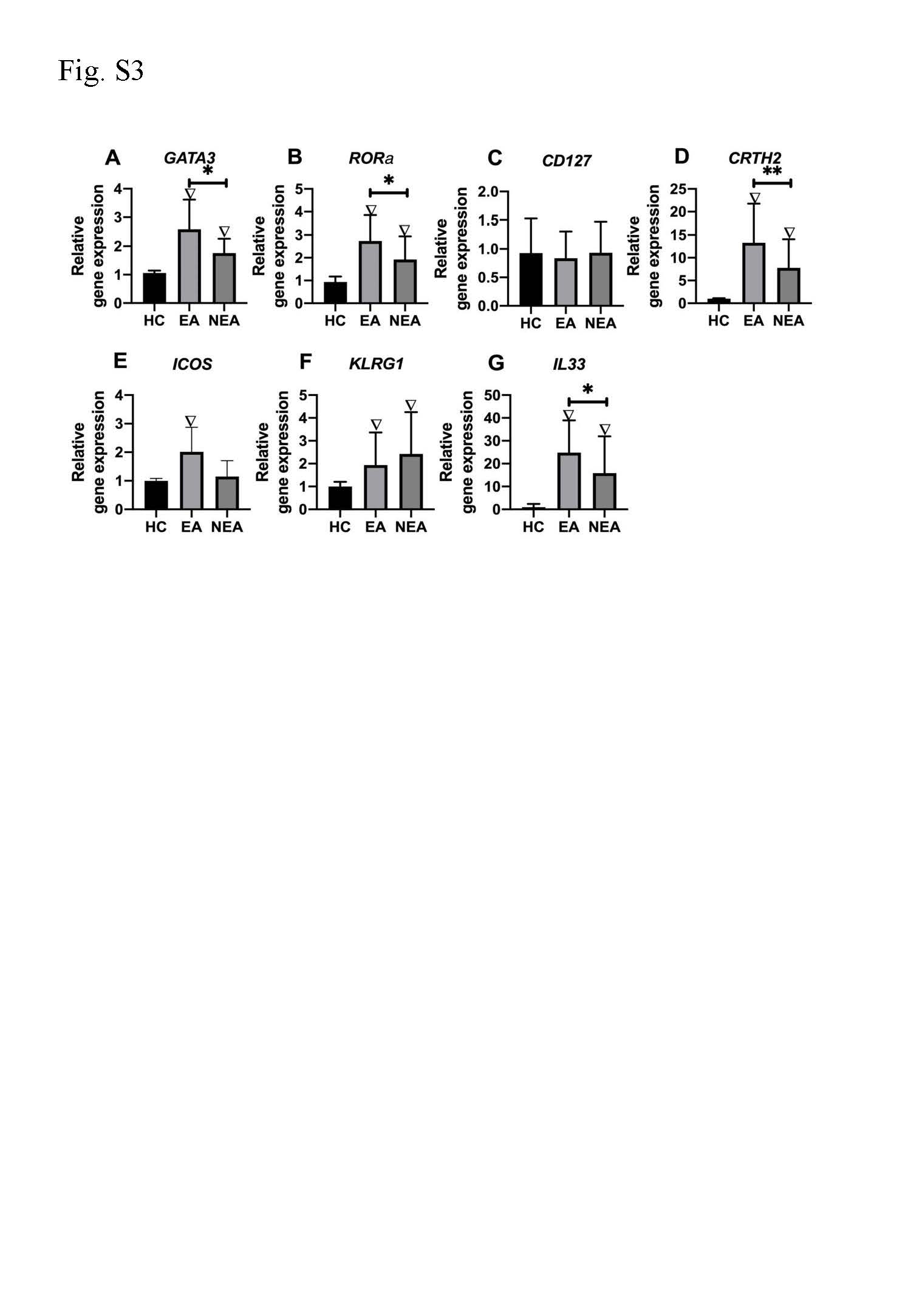

Supplement: Supplementary file 4 — Supplementary Material [file CLT2-13-e12265-s002.jpg]

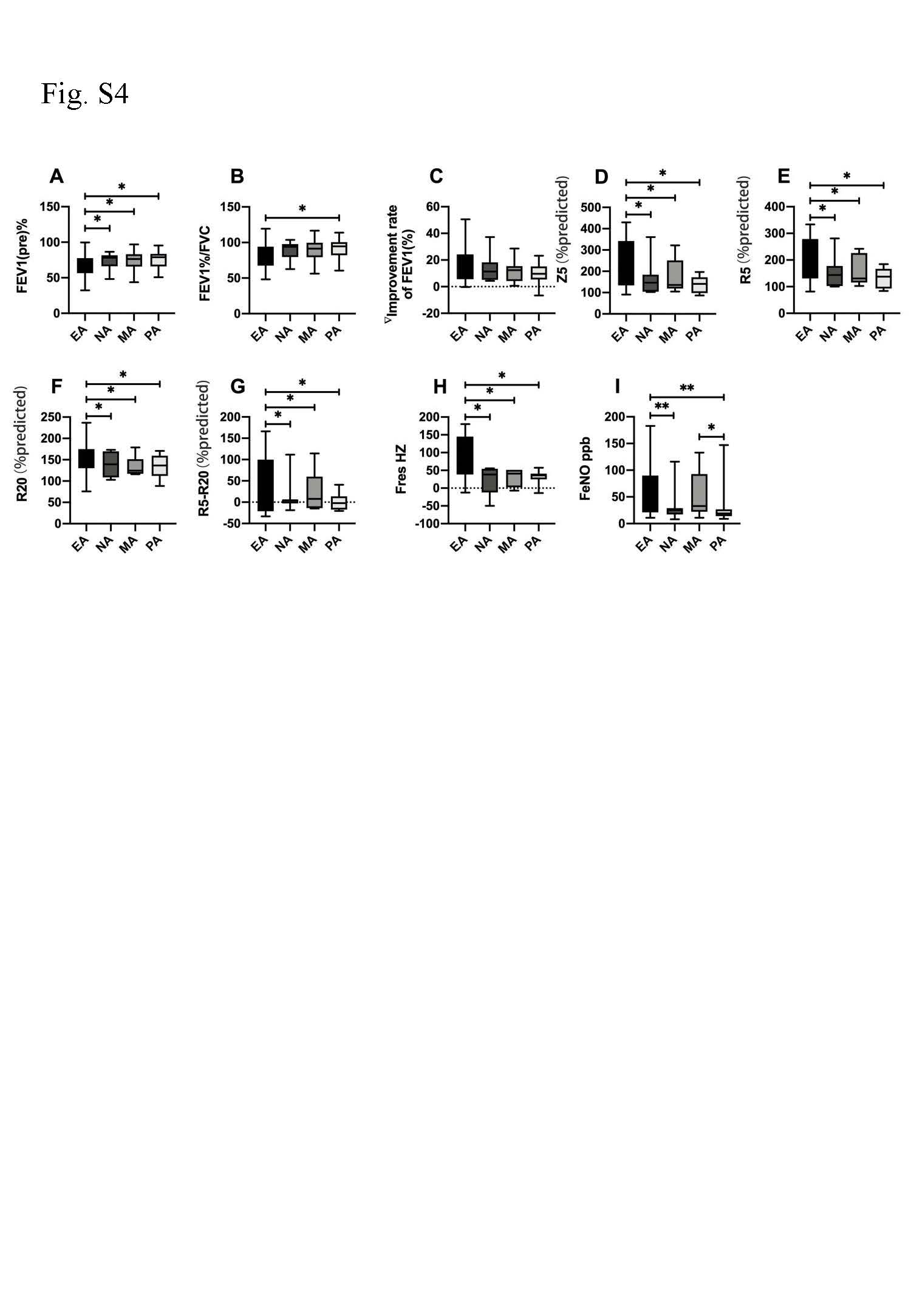

Supplement: Supplementary file 5 — Supplementary Material [file CLT2-13-e12265-s008.jpg]

## Slide 1
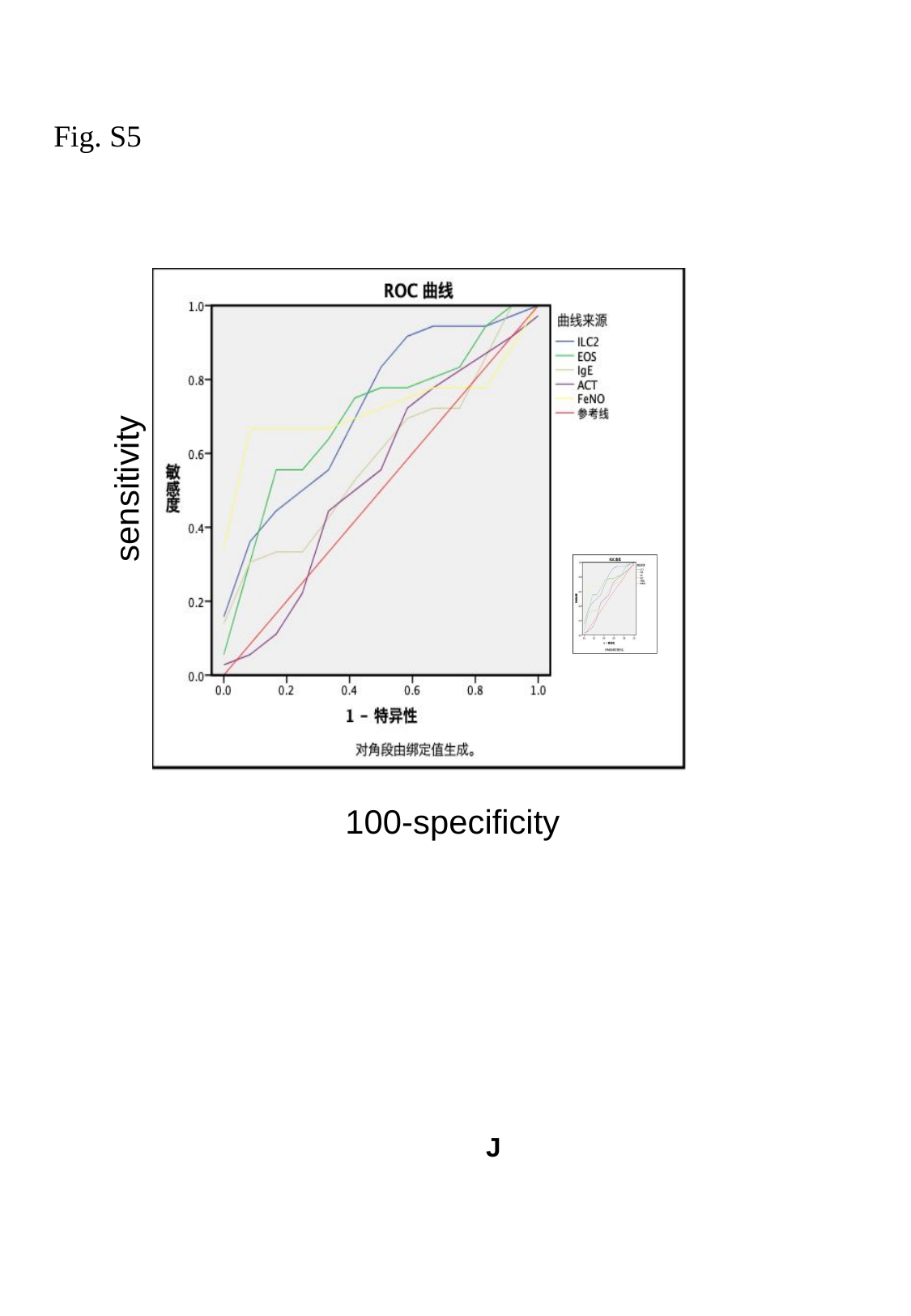

Fig. S5
sensitivity
100-specificity
J

Supplement: Supplementary file 6 — Supplementary Material [file CLT2-13-e12265-s007.pptx]

## Slide 1
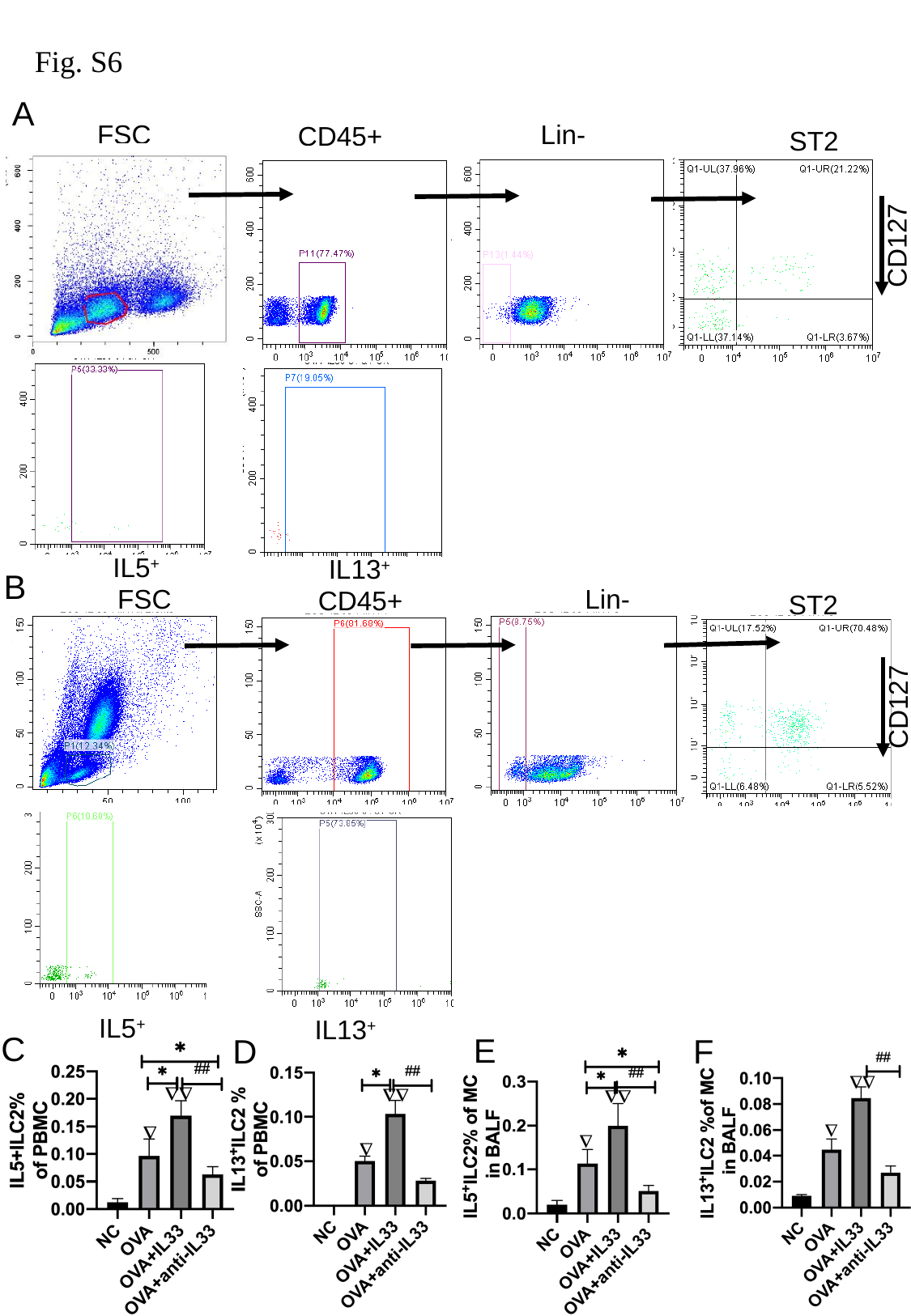

Fig. S6
A
FSC
Lin-
CD45+
ST2
CD127
IL5+
IL13+
B
FSC
Lin-
CD45+
ST2
CD127
IL5+
IL13+
C
E
D
F

Supplement: Supplementary file 7 — Supplementary Material [file CLT2-13-e12265-s006.pptx]

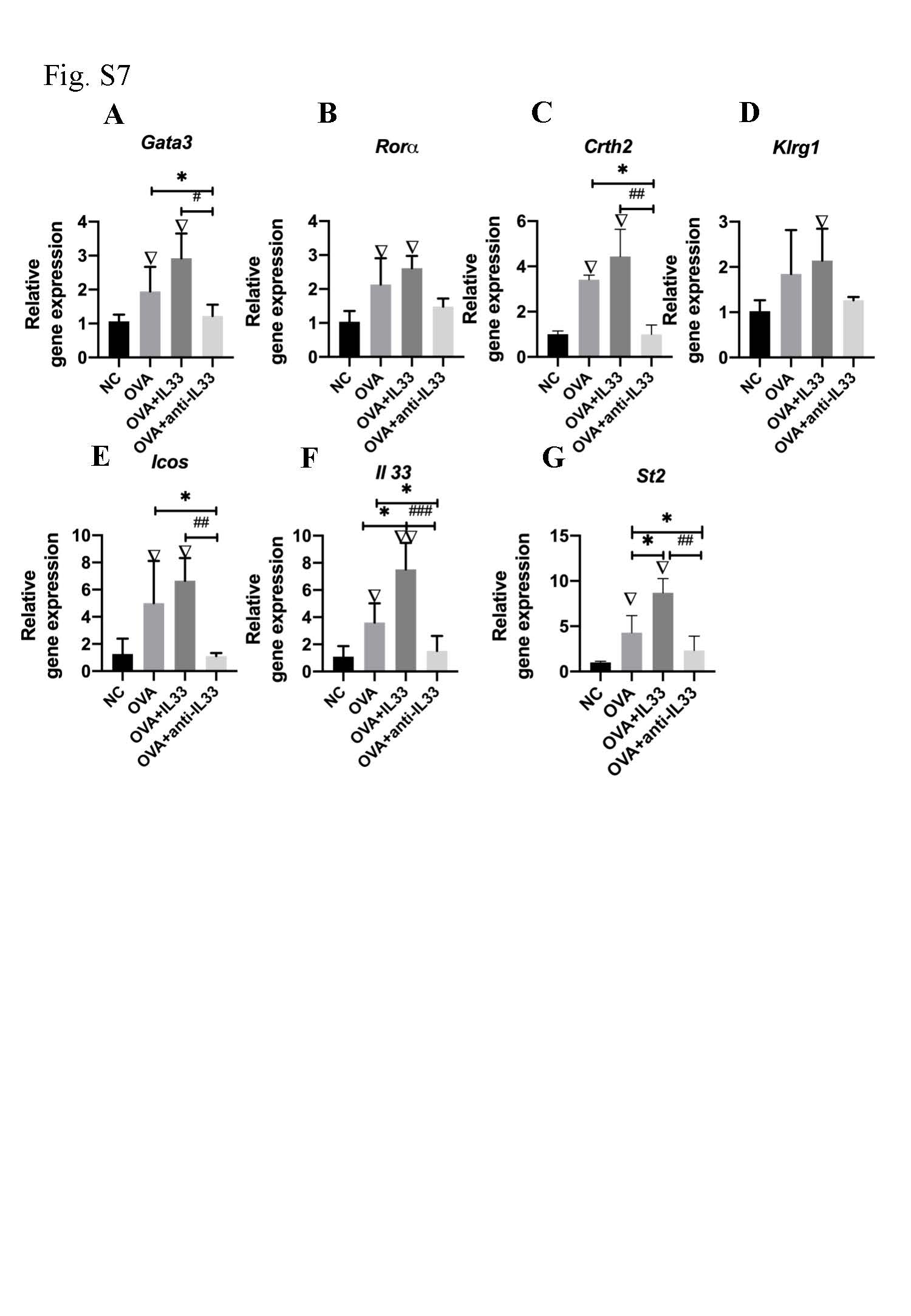

Supplement: Supplementary file 8 — Supplementary Material [file CLT2-13-e12265-s011.jpg]

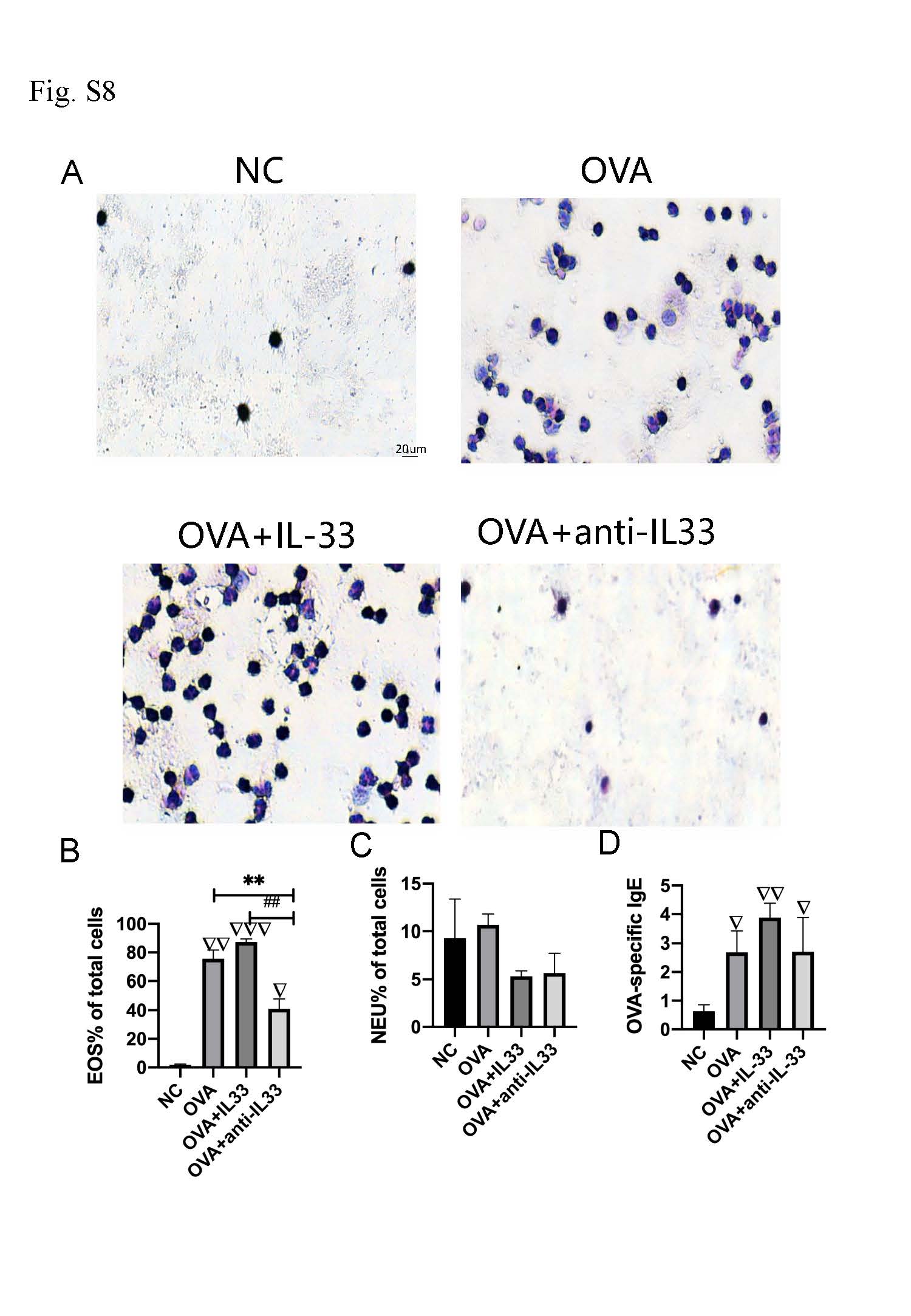

Supplement: Supplementary file 9 — Supplementary Material [file CLT2-13-e12265-s010.jpg]

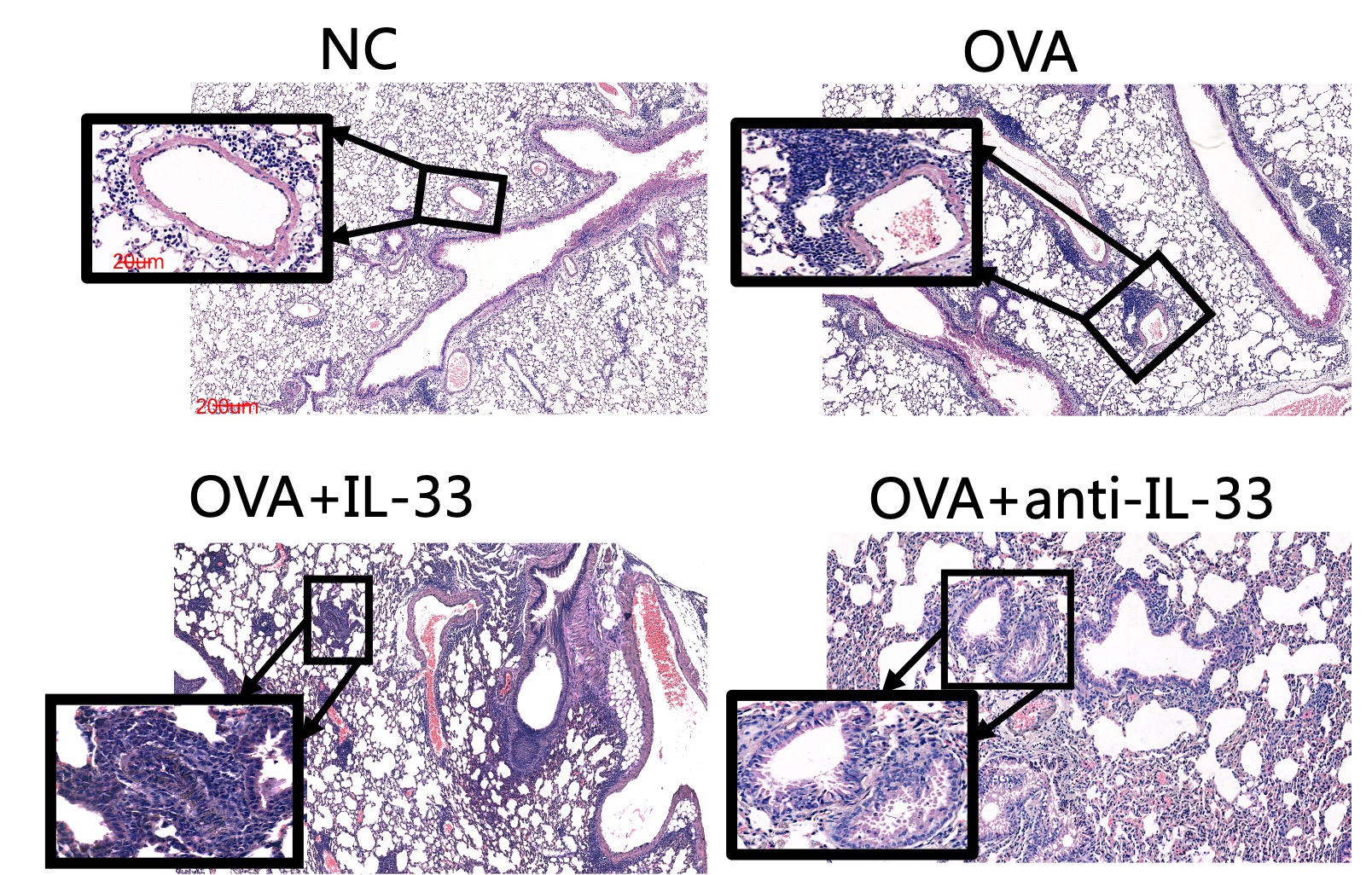

Supplement: Supplementary file 10 — Supplementary Material [file CLT2-13-e12265-s001.jpg]

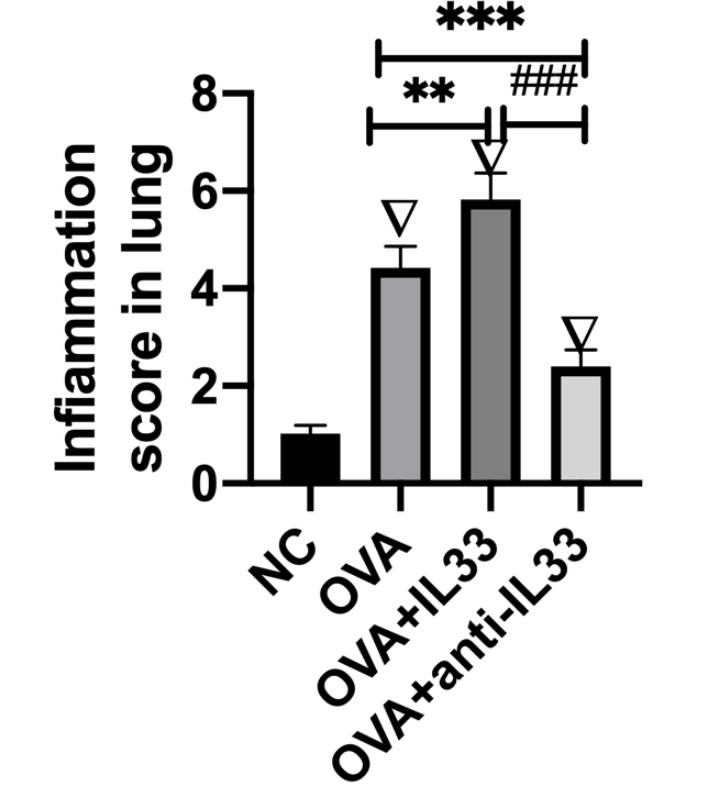

Supplement: Supplementary file 11 — Supplementary Material [file CLT2-13-e12265-s005.jpg]
